# Endothelial AGGF1 Deficiency Causes Mitochondrial Dysfunction and Contributes to Age‐Elevated Blood Pressure

**DOI:** 10.1111/acel.70652

**Published:** 2026-08-03

**Authors:** Weixin Lv, Xiaojuan Zhong, Qiang Yuan, Xueting Gong, Ya Zhao, Shilin Zhang, Andong Wu, Ming Wan, Xueer Li, Yangyi Zheng, Jiankun Liu, Bingbing Zhou, Yuanzheng Zhu, Limin Zhao, Qiquan Wang, Yang Xiang, Xiao‐Li Tian

**Affiliations:** ^1^ Aging and Vascular Diseases, Human Aging Research Institute (HARI) and School of Life Science Nanchang University, and Jiangxi Province Key Laboratory of Aging and Disease Nanchang Jiangxi China; ^2^ Human Aging Research Institute (HARI) and School of Life Science Nanchang University, and Jiangxi Province Key Laboratory of Aging and Disease Nanchang Jiangxi China; ^3^ Metabolic Control and Aging, Human Aging Research Institute (HARI) and School of Life Science Nanchang University, and Jiangxi Province Key Laboratory of Aging and Disease Nanchang Jiangxi China

**Keywords:** AGGF1, blood pressure, early vascular aging, endothelial cell, mitochondrial dysfunction, SESN2

## Abstract

Endothelial cells are critical in the regulation of blood pressure. We previously demonstrated AGGF1 was crucial for maintaining endothelial cell function. This study aims to examine whether AGGF1 regulates blood pressure. Here, we found that AGGF1 expression was inversely correlated with blood pressure with age. In male mice, endothelial‐specific loss of function of *Aggf1* (*Aggf1* ^*flox/flox*^ */Tie2‐Cre* ^+^) significantly increased blood pressure, whereas endothelial‐specific human *AGGF1* overexpression (TGM(Tie2‐hAGGF1)) suppressed age‐elevated blood pressure. The endothelial‐specific loss of AGGF1 accelerated early vascular aging, characterized by increased arterial stiffness, impaired endothelium‐dependent vasorelaxation, reduced eNOS phosphorylation, and augmented ROS production, whereas AGGF1 overexpression rescued these phenotypic changes. Mechanistically, we found that nuclear‐localized AGGF1 bound to the SESN2 promoter and enhanced its transcription. This upregulation of SESN2 attenuated mitochondrial dysfunction, including respiratory dysfunction and elevated mitochondrial ROS, and ultimately led to increased p‐eNOS levels and improved endothelial function. Furthermore, SESN2 overexpression rescued early vascular aging and reduced blood pressure in *Aggf1* ^*flox/flox*^ */Tie2‐Cre* ^+^ mice, while SESN2 knockdown attenuated the beneficial vascular effects of AGGF1. Together, we demonstrate that endothelial AGGF1/SESN2/p‐eNOS is a novel and important signaling axis in the maintenance of blood pressure. Age suppresses the AGGF1/SESN2/p‐eNOS signaling cascade, leading to an elevation of blood pressure. Our study provides new insights into age‐elevated blood pressure in male mice and suggests AGGF1 as a potentially valuable target for prehypertension intervention in the elderly male population.

## Graphical abstract

Endothelial AGGF1 deficiency contributes to early vascular aging and age‐elevated blood pressure via inhibiting the SESN2/p‐eNOS pathway. Age‐dependent AGGF1 attenuation reduces its binding to the SESN2 promoter. SESN2 downregulation induces mitochondrial respiratory dysfunction and elevated mitochondrial ROS, thereby reducing eNOS phosphorylation. These effects contribute to accelerated early vascular aging and age‐elevated blood pressure.

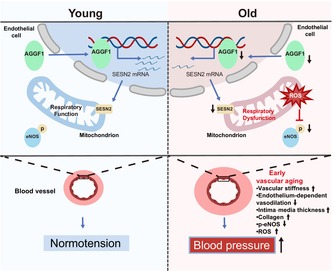

## 1 Introduction

Hypertension is a common age‐related cardiovascular risk factor and a leading underlying cause of mortality and disability in the elderly (Benetos et al. 2019; Kannel 2000; Long et al. 2022; Susic and Frohlich 2008). Among individuals aged 60 and above, hypertension prevalence exceeds 67.8% (Ma et al. 2021; Xing et al. 2020). Similarly, blood pressure in aged mice is also significantly elevated (Feng et al. 2020; Kong et al. 2020; Liu et al. 2025; Wirth et al. 2016). Prehypertension (blood pressure 120–139/80–89 mmHg) represents an intermediate state between normotension and hypertension, and is associated with an increased risk of both incident hypertension and cardiovascular events (Egan and Stevens‐Fabry 2015; Huang et al. 2015; Zhao et al. 2021). Prehypertension results from the interplay of genetic and environmental factors (Davis et al. 2012; Zhao, Du, et al. 2025). Its risk can be mitigated by modifying environmental factors such as an unhealthy diet, lack of exercise, weight gain, and alcohol consumption (Albarwani et al. 2014).

Endothelial dysfunction is a critical initiating step in the development of prehypertension and hypertension (Dikalova et al. 2024; Li et al. 2019; Zhao et al. 2021). During aging, endothelial cells, localized in the innermost vascular layer, are chronically exposed to stressors such as high glucose, lipids, and shear forces (Zhang et al. 2024; Zhang et al. 2025). This exposure results in endothelial damage and premature senescence (Wang et al. 2018; Zhu et al. 2022). The dysfunctional endothelial cells exhibit mitochondrial dysfunction characterized by excessive mitochondrial reactive oxygen species (mtROS) production, leading to reduced endothelial nitric oxide synthase (eNOS) activity, impaired endothelium‐dependent vasodilation, consequently resulting in early vascular aging and blood pressure elevation (Majerczak et al. 2019; Sun 2015; Ungvari et al. 2018).

Angiogenic factor with G‐patch and FHA domains 1 (AGGF1, also known as VG5Q) is an emerging angiogenic factor associated with Klippel‐Trenaunay syndrome (KTS) (Tian et al. 2004). Our early study showed that AGGF1 has paracrine or autocrine effects as it can be secreted (Tian et al. 2004). However, we found later that AGGF1, adapted for distinct functional requirements, shuttles between nucleocytoplasmic compartments (Zhang et al. 2021). The nuclear localization of AGGF1 is suggestive of its regulatory role in cellular transcription. AGGF1 also acts as a critical protective factor for vessels. We previously demonstrated that AGGF1 inhibits TNF‐α‐induced inflammatory responses in endothelial cells by suppressing the ERK/NF‐κB pathway (Hu et al. 2013). In addition, AGGF1 reduces vascular permeability by inhibiting VE‐cadherin phosphorylation (Zhang et al. 2016). Notably, a study showed that AGGF1 expression was decreased in hypertensive patients (Gao et al. 2026). Although this observation suggests a potential involvement of AGGF1 in blood pressure regulation, the direct causal link remains to be established.

In this study, we employed male mouse models with endothelial‐specific knockout or overexpression of AGGF1 to investigate the effect of AGGF1 on blood pressure. We show that age decreases vascular expression of AGGF1, which in turn elevates blood pressure and accelerates early vascular aging, and identify AGGF1/SESN2/p‐eNOS as a novel signaling axis contributing to blood pressure regulation in male mice.

## 2 Results

### 2.1 Age‐Attenuated Endothelial AGGF1 Expression Elevates Blood Pressure, Whereas AGGF1 Overexpression Rescues This Phenotype

**FIGURE 1:**
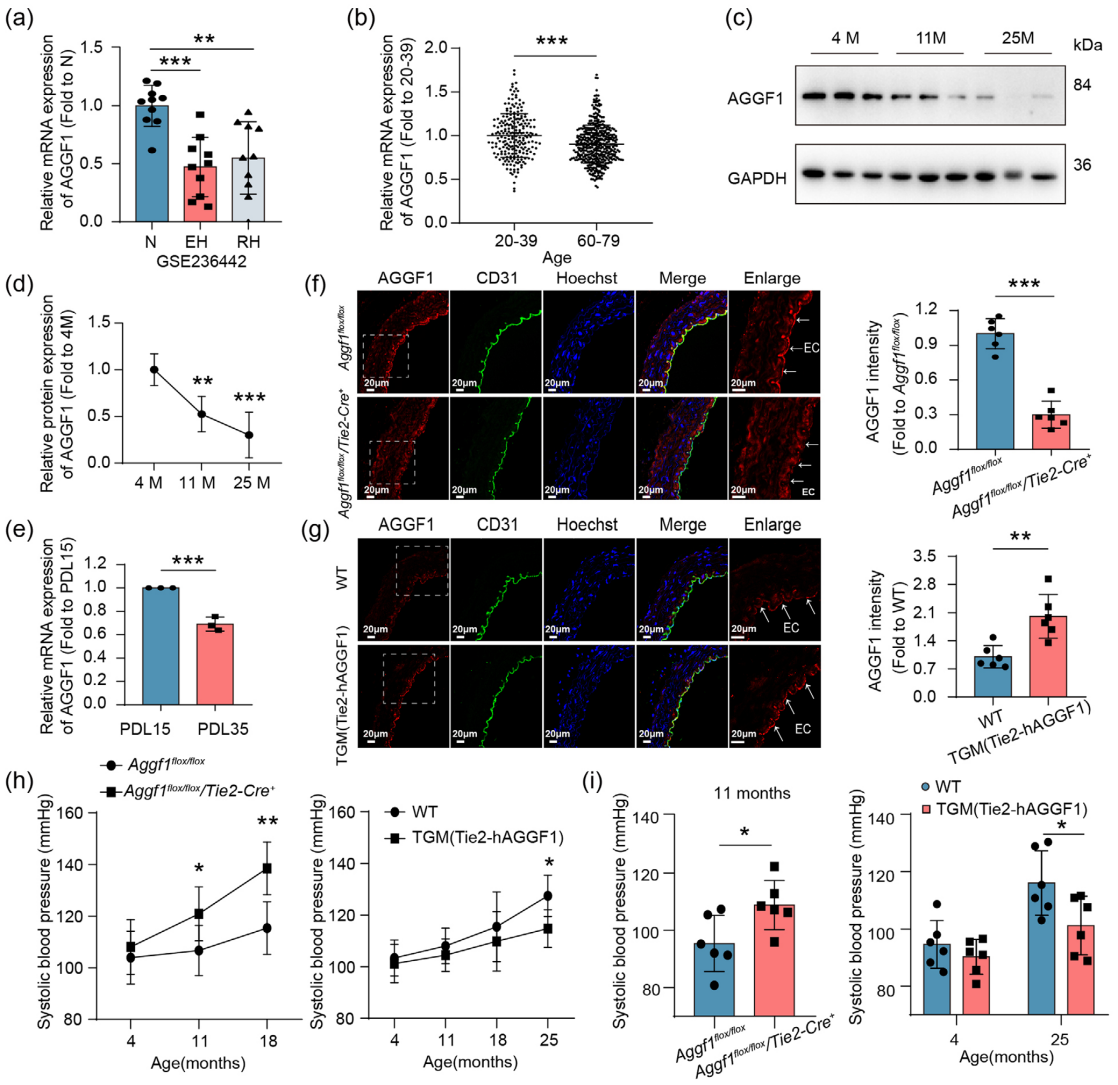
Age‐attenuated endothelial AGGF1 expression elevates blood pressure, whereas AGGF1 overexpression rescues this phenotype. (a) AGGF1 mRNA levels in peripheral blood from subjects with resistant hypertension (RH, *n* = 10 per group), subjects with essential hypertension (EH, *n* = 10 per group), and normotensive subjects (*N*, *n* = 10 per group) (GEO: GSE236442). One‐way ANOVA. (b) AGGF1 expression in human vascular tissues from young (20–39 years) compared with that from aged (60–79 years) donors. Two‐tailed Student's *t*‐test. (c, d) Western blot analysis of AGGF1 protein expression in the aortas of young (4‐month‐old), middle‐aged (11‐month‐old), and aged (25‐month‐old) mice (*n* = 6 per group). One‐way ANOVA. (e) AGGF1 mRNA levels in HUVECs at population doubling levels (PDL) 15 and 35 were analyzed by qRT‐PCR (*n* = 3 per group, 3 independent experiments). Two‐tailed Student's *t*‐test. (f, g) Immunofluorescence staining for AGGF1 (red) and CD31 (green) in the aorta of *Aggf1* ^*flox/flox*^ */Tie2‐Cre* ^+^ and TGM(Tie2‐hAGGF1) mice (*n* = 6 per group), scale bar = 20 μm. Two‐tailed Student's *t*‐test. (h) SBP in *Aggf1* ^*flox/flox*^ */Tie2‐Cre* ^+^ (*n* = 8 per group at 4, 11, and 18 months) and TGM(Tie2‐hAGGF1) mice (*n* = 8 per group at 4, 11, 18, and 25 months) at the indicated ages, as assessed by the tail‐cuff method. Two‐way ANOVA. (i) SBP measured by carotid artery cannulation in mice at the indicated ages (*n* = 6 per group). Two‐tailed Student's *t*‐test and one‐way ANOVA. Data are presented as mean ± SD. *p < 0.05, **p < 0.01, ***p < 0.001.

To investigate the role of AGGF1 in age‐elevated blood pressure, we first analyzed AGGF1 mRNA levels in hypertensive patients using the Gene Expression Omnibus (GEO) database. The dataset (GSE236442) comprised peripheral blood samples from 10 patients with resistant hypertension (RH), 10 patients with essential hypertension (EH), and 10 healthy individuals (N). The results revealed that AGGF1 mRNA levels were significantly downregulated in the whole blood of hypertensive patients (Figure 1a). Subsequently, we retrieved the human vascular database (ADEIP) and found that AGGF1 expression in human vasculature decreased significantly during aging (Figure 1b). Consistently, Western blot analysis of arterial lysates revealed an age‐dependent decline in vascular AGGF1 levels in mice (Figure 1c,d). In vitro, AGGF1 expression was markedly reduced in senescent endothelial cells (Figure 1e), suggesting its role in endothelial senescence associated with age‐related increases in blood pressure.

Next, we generated endothelial‐specific *Aggf1* knockout mice (*Aggf1* ^*flox/flox*^ */Tie2‐Cre* ^+^) (Figure S1a) and endothelial‐specific human *AGGF1* overexpression mice (TGM(Tie2‐hAGGF1)) (Figure S1b). AGGF1 levels in the aorta of *Aggf1* ^*flox/flox*^ */Tie2‐Cre* ^+^ mice were significantly reduced (Figure 1f), while AGGF1 levels were significantly increased in the aorta of TGM(Tie2‐hAGGF1) mice (Figure 1g). Mouse blood pressure was measured using the tail‐cuff method. We found that control C57BL/6 mice maintained relatively stable systolic blood pressure (SBP) until advanced age (25‐month‐old), after which it increased significantly (Figure 1h). SBP in *Aggf1* ^*flox/flox*^ */Tie2‐Cre* ^+^ mice did not differ significantly from that of age‐matched controls at 4 months, but exhibited a statistically significant elevation at 11 and 18 months (Figure 1h,i). In contrast, 25‐month‐old TGM(Tie2‐hAGGF1) mice exhibited significantly lower SBP (Figure 1h), which was further confirmed by carotid artery cannulation (Figure 1i).

### 2.2 Endothelial‐Specific AGGF1 Knockout Promotes Early Vascular Aging in Mice, Whereas AGGF1 Overexpression Rescues Vascular Aging

**FIGURE 2:**
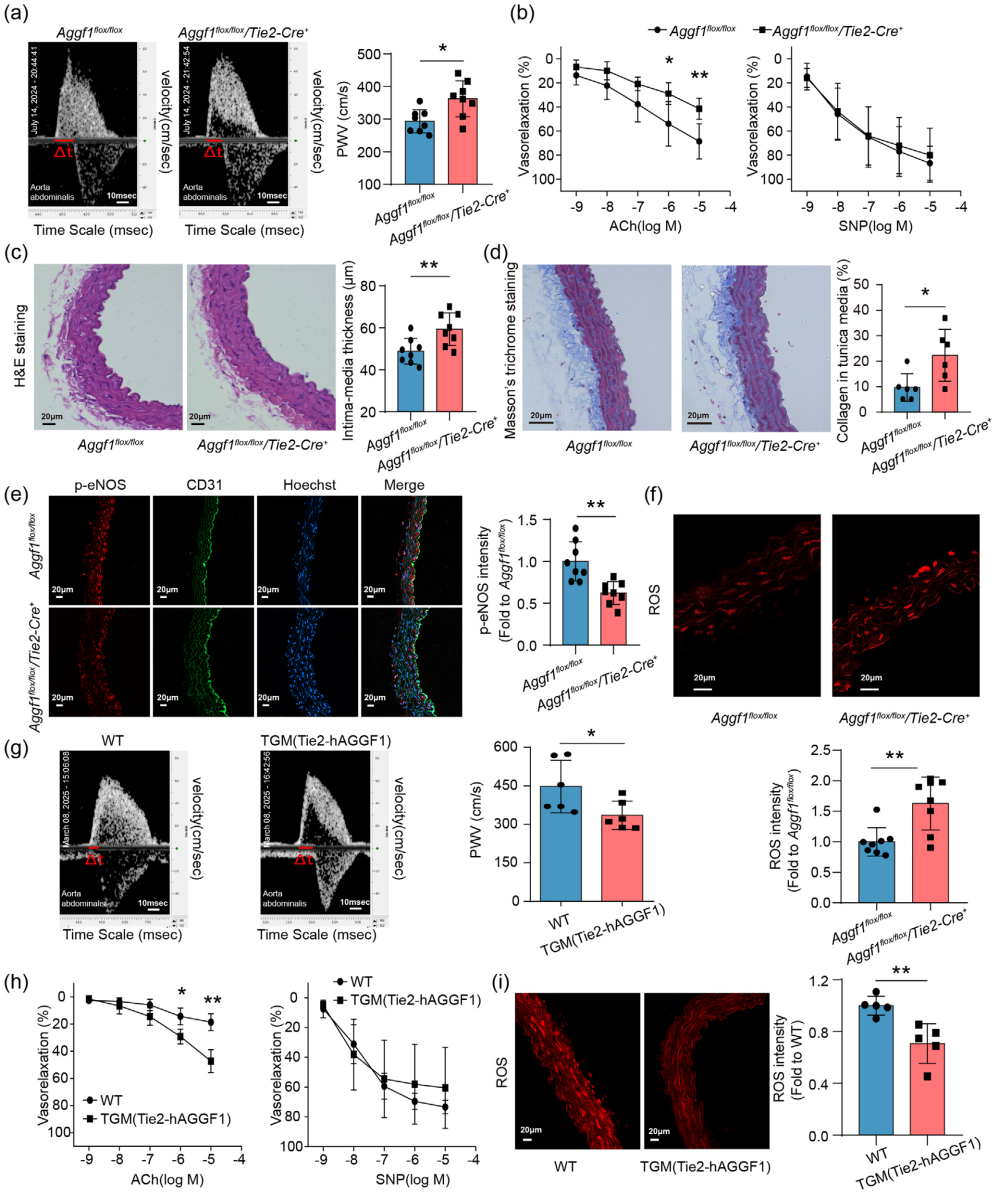
Endothelial AGGF1 deficiency promotes early vascular aging, whereas its overexpression rescues vascular aging. Analysis of the aorta in 11‐month‐old *Aggf1* ^*flox/flox*^ */Tie2‐Cre* ^+^ mice (a–f) and 25‐month‐old TGM(Tie2‐hAGGF1) mice (g‐i). (a, g) Representative images and quantification of aortic PWV in *Aggf1* ^*flox/flox*^ */Tie2‐Cre* ^+^ mice (*n* = 8 per group) and TGM(Tie2‐hAGGF1) mice (*n* = 6 per group). Two‐tailed Student's *t*‐test. (b, h) Endothelium‐dependent vasorelaxation responses to ACh and endothelium‐independent responses to SNP were measured in aortic rings of *Aggf1* ^*flox/flox*^ */Tie2‐Cre* ^+^ mice (*n* = 8 per group) or TGM(Tie2‐hAGGF1) mice (*n* = 5 per group). Two‐way ANOVA. (c) Representative H&E staining and intima‐media thickness quantification (*n* = 8 per group), scale bar = 20 μm. Two‐tailed Student's *t*‐test. (d) Representative micrographs and quantification of Masson's trichrome staining of the aorta (*n* = 6 per group), scale bar = 20 μm. Two‐tailed Student's *t*‐test. (e) Immunofluorescence staining for p‐eNOS (red), CD31 (green), and Hoechst (blue) in the aortas (*n* = 8 per group), scale bar = 20 μm. Two‐tailed Student's *t*‐test. (f, i) Fluorescence staining of ROS (red) in the aorta of *Aggf1* ^*flox/flox*^ */Tie2‐Cre* ^+^ mice (*n* = 8 per group) or TGM(Tie2‐hAGGF1) mice (*n* = 5 per group), scale bar = 20 μm. Two‐tailed Student's *t*‐test. Data are presented as mean ± SD. *p < 0.05, **p < 0.01.

Doppler ultrasound analysis revealed increased arterial stiffness in *Aggf1* ^*flox/flox*^ */Tie2‐Cre* ^+^ mice, as evidenced by increased pulse wave velocity (PWV) (Figure 2a). Conversely, endothelial‐specific AGGF1 overexpression decreased arterial stiffness in aged mice (Figure 2g). Functional assessments revealed impaired endothelium‐dependent vasorelaxation of the aorta in *Aggf1* ^*flox/flox*^ */Tie2‐Cre* ^+^ mice, whereas endothelium‐independent relaxation was unaffected (Figure 2b). Notably, we observed a marked impairment of endothelium‐dependent vasodilation in aged mice without affecting endothelium‐independent relaxation (Figure S2a,b). This age‐related endothelial dysfunction was rescued by endothelial‐specific AGGF1 overexpression (Figure 2h). Morphological analysis demonstrated aortic wall thickening and increased collagen deposition in *Aggf1* ^*flox/flox*^ */Tie2‐Cre* ^+^ mice (Figure 2c,d).

At the molecular level, *Aggf1* ^*flox/flox*^ */Tie2‐Cre* ^+^ mice exhibited decreased p‐eNOS levels (Figure 2e) and elevated reactive oxygen species (ROS) production (Figure 2f), while aged TGM(Tie2‐hAGGF1) mice showed reduced oxidative stress (Figure 2i). These findings establish AGGF1 as a key protective factor against early vascular aging.

### 2.3 AGGF1 Delays Endothelial Cell Senescence In Vitro

**FIGURE 3:**
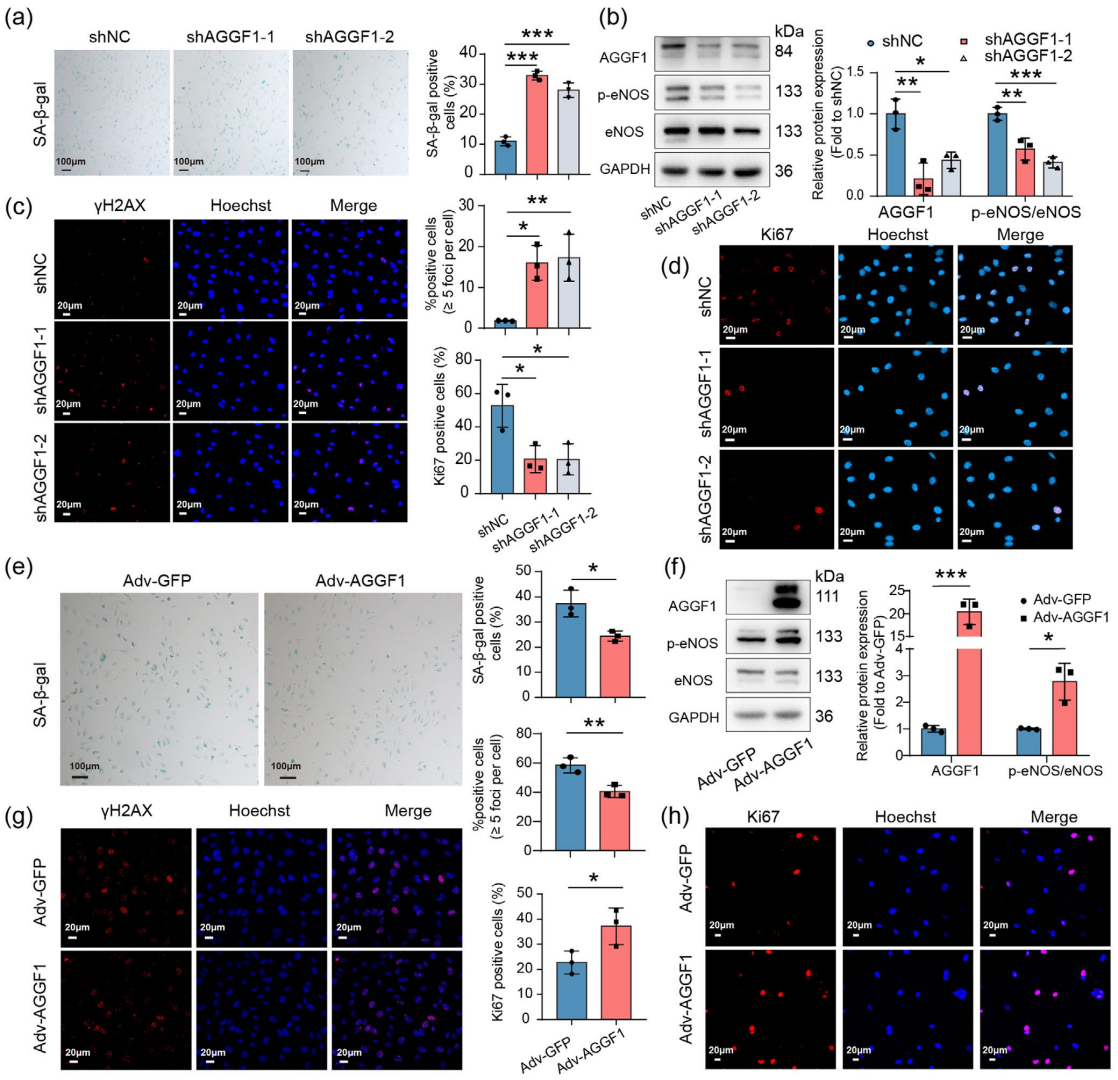
AGGF1 delays endothelial cell senescence in vitro. HUVECs were infected with lentivirus expressing shRNA against AGGF1 (shAGGF1) or negative control (shNC) (a–d). Alternatively, HUVECs were infected with adenovirus expressing AGGF1 (Adv‐AGGF1) or control virus (Adv‐GFP), and then treated with DOX (e‐h). (a, e) Representative images and quantification of SA‐β‐gal positive cells. (*n* = 3 per group, 3 independent experiments), scale bar = 100 μm. One‐way ANOVA and two‐tailed Student's *t*‐test. (b, f) Western blot analysis of AGGF1, eNOS, and p‐eNOS (Ser1177) levels (*n* = 3 per group, 3 independent experiments). One‐way ANOVA and two‐tailed Student's *t*‐test. (c, g) Immunofluorescence staining for γH2AX (red) and Hoechst (blue) in HUVECs (*n* = 3 per group, 3 independent experiments), scale bar = 20 μm. One‐way ANOVA and two‐tailed Student's *t*‐test. (d, h) Immunofluorescence staining for Ki67 (red) and Hoechst (blue) in HUVECs (*n* = 3 per group, 3 independent experiments), scale bar = 20 μm. One‐way ANOVA and two‐tailed Student's *t*‐test. Data are presented as mean ± SD. *p < 0.05, **p < 0.01, ***p < 0.001.

To determine whether AGGF1 deficiency induces endothelial senescence, two shRNAs (shAGGF1‐1, shAGGF1‐2) were used to knock down AGGF1 in human umbilical vein endothelial cells (HUVECs). AGGF1 mRNA expression was significantly reduced by shRNAs (Figure S3a). Subsequent evaluation of senescence markers in AGGF1‐knockdown HUVECs revealed a higher percentage of senescence‐associated β‐galactosidase (SA‐β‐gal) positive cells (Figure 3a), reduced p‐eNOS levels (Figure 3b), and decreased nitric oxide (NO) concentration (Figure S4a). Moreover, immunofluorescence analysis revealed increased phosphorylated histone H2AX (γH2AX) levels (Figure 3c) and a decreased proportion of Ki67 positive cells (Figure 3d) in AGGF1‐knockdown HUVECs. Additionally, AGGF1 knockdown significantly upregulated IL‐6 mRNA levels (Figure S4b).

To investigate whether AGGF1 overexpression delays endothelial senescence, we transduced HUVECs with adenoviral or lentiviral vectors expressing AGGF1 (Figure S3b,c) and subsequently treated the cells with doxorubicin (DOX) to induce senescence. DOX treatment significantly increased the percentage of SA‐β‐gal positive cells (Figure S5a), reduced p‐eNOS levels (Figure S5b), elevated γH2AX levels (Figure S5c), decreased the proportion of Ki67 positive cells (Figure S5d), and enhanced mtROS production (Figure S5e). Notably, AGGF1 overexpression significantly delayed DOX‐induced endothelial cell senescence, as evidenced by a reduced percentage of SA‐β‐gal positive cells (Figure 3e), increased p‐eNOS levels (Figure 3f), reduced γH2AX levels (Figure 3g), and an increased proportion of Ki67 positive cells (Figure 3h). These findings suggest that AGGF1 delays endothelial cell senescence in HUVECs.

### 2.4 AGGF1 Regulates Mitochondrial Function and Sestrin2 (SESN2) Transcription

**FIGURE 4:**
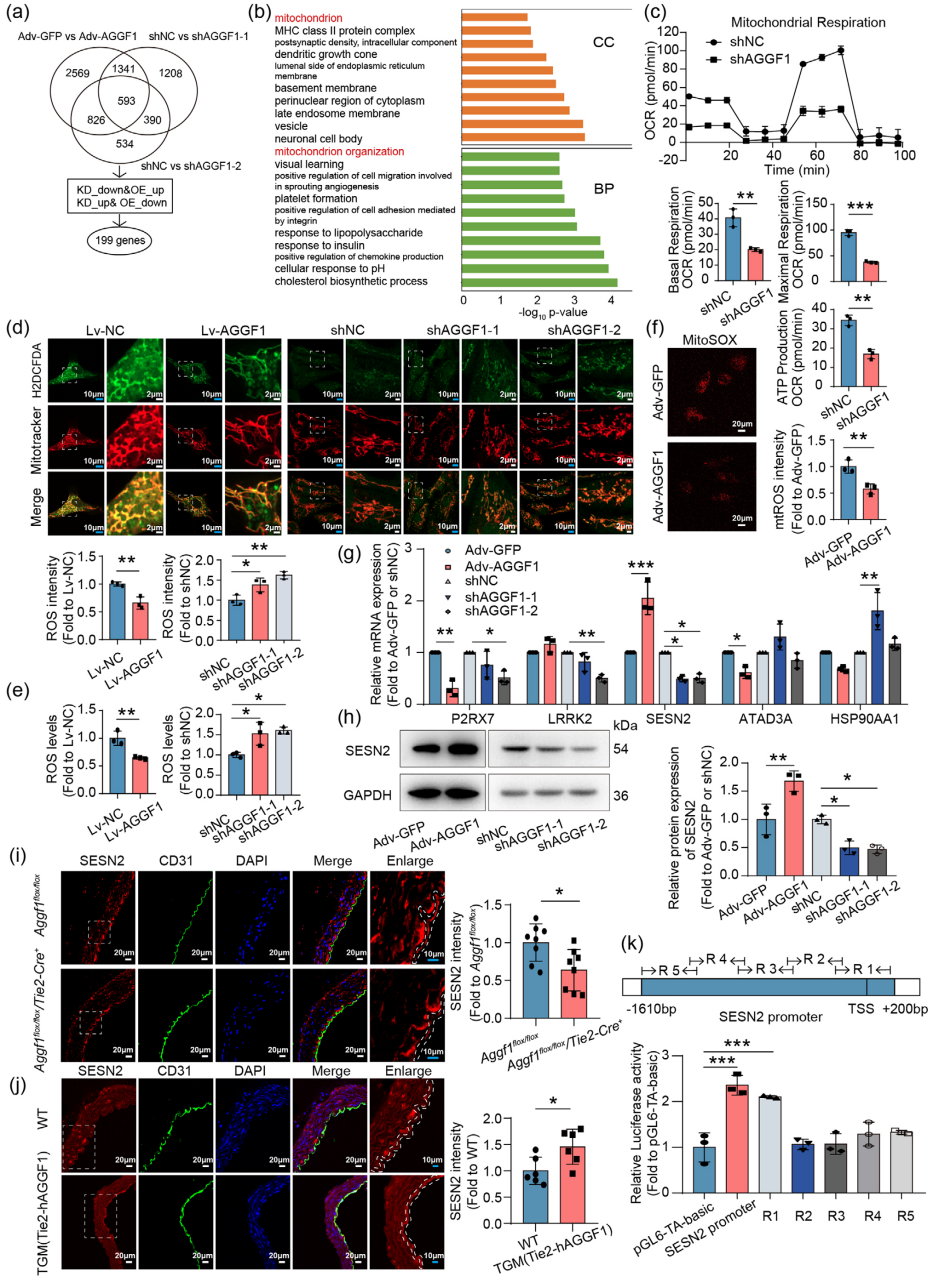
AGGF1 regulates mitochondrial function and SESN2 transcription. (a) Venn diagram showing the 199 genes co‐expressed with AGGF1 across both AGGF1 overexpression and knockdown datasets. (b) GO enrichment analysis identified the top 10 biological processes (BP) and cellular components (CC) among the 199 genes. (c) OCR in HUVECs was measured using the Seahorse XF Cell Mitochondrial Stress Test (*n* = 3 per group, 3 independent experiments). Two‐tailed Student's *t*‐test. (d) Fluorescence staining of ROS (green, H_2_DCFDA) and mitochondria (red, MitoTracker) in HUVECs, with quantitative analysis of relative H_2_DCFDA fluorescence intensity (*n* = 3 per group, 3 independent experiments), blue scale bar = 10 μm, white scale bar = 2 μm. Two‐tailed Student's *t*‐test and one‐way ANOVA. (e) ROS production was measured using a microplate reader with H_2_DCFDA (*n* = 3 per group, 3 independent experiments). Two‐tailed Student's *t*‐test and one‐way ANOVA. (f) Representative images of mtROS detected by MitoSOX staining, with quantitative analysis of relative fluorescence intensity (*n* = 3 per group, 3 independent experiments), scale bar = 20 μm. Two‐tailed Student's *t*‐test. (g) Expression levels of senescence‐associated genes in HUVECs measured by qRT‐PCR (*n* = 3 per group, 3 independent experiments). One‐way ANOVA. (h) Western blot analysis of SESN2 protein expression in HUVECs (*n* = 3 per group, 3 independent experiments). One‐way ANOVA. (i, j) Immunofluorescence staining for SESN2 (red), CD31 (green), and DAPI (blue) in the aorta of *Aggf1* ^*flox/flox*^ */Tie2‐Cre* ^+^ mice (*n* = 8 per group) or TGM(Tie2‐hAGGF1) mice (*n* = 6 per group), white scale bar = 20 μm, blue scale bar = 10 μm. Two‐tailed Student's *t*‐test. (k) Schematic diagram of the human SESN2 promoter region (−1610 to +200 bp) and luciferase reporter assay in HEK293T cells (*n* = 3 per group, 3 independent experiments). One‐way ANOVA. Data are presented as mean ± SD. *p < 0.05, **p < 0.01, ***p < 0.001.

To investigate the mechanism by which AGGF1 regulates endothelial cellular senescence, we performed RNA sequencing of HUVECs following AGGF1 overexpression or knockdown. Among the three datasets, 593 overlapping genes were identified. Of these, 199 were retained because they were differentially co‐expressed with AGGF1, suggesting they are potential targets (Figure 4a). Gene Ontology (GO) enrichment analysis indicated that AGGF1 modulates mitochondrial‐related pathways (Figure 4b), which are known to contribute to cellular senescence (Miwa et al. 2022). To further explore the effects of AGGF1 on mitochondria, we analyzed the oxygen consumption rate (OCR) of HUVECs and found that AGGF1 knockdown impaired maximal, basal, and ATP‐linked mitochondrial respiration (Figure 4c). Furthermore, AGGF1 overexpression reduced mtROS levels, whereas its knockdown increased them. This was evidenced by co‐localization imaging with H_2_DCFDA and MitoTracker (Figure 4d,e), and further verified using MitoSOX Red staining (Figure 4f).

Among the identified mitochondrial pathway‐related genes (Figure S6), we validated the expression of senescence‐associated genes P2RX7, LRRK2, SESN2, ATAD3A, and HSP90AA1 using quantitative real‐time polymerase chain reaction (qRT‐PCR). AGGF1 most significantly regulated SESN2 mRNA expression (Figure 4g). At the protein level, AGGF1 overexpression increased SESN2 expression, whereas AGGF1 knockdown reduced its expression (Figure 4h). Consistent with these findings, immunofluorescence staining confirmed reduced SESN2 levels in aortic endothelial cells of *Aggf1* ^*flox/flox*^ */Tie2‐Cre* ^+^ mice, while SESN2 expression was increased in aortic endothelial cells of TGM(Tie2‐hAGGF1) mice (Figure 4i,j). Furthermore, we identified a positive correlation between SESN2 expression and AGGF1 expression in vascular tissues of the elderly by analyzing the ADEIP database (Figure S7a). Additionally, SESN2 expression decreased with age in mouse aortic endothelium (Figure S7b).

AGGF1 exhibited nuclear localization in quiescent endothelial cells (Figure S8), indicating its intrinsic capacity for transcriptional regulation. Importantly, although AGGF1 can be secreted, functional assays using a nuclear localization signal (NLS)‐deficient mutant (AGGF1‐ΔNLS) confirmed that nuclear AGGF1 is essential for delaying endothelial cell senescence (Figure S9a,b). Luciferase reporter assays revealed that AGGF1 significantly enhanced SESN2 promoter activity (Figure 4k), suggesting that AGGF1 acts as an endothelial cell transcriptional regulator that activates SESN2 expression through promoter binding. To map AGGF1‐binding sites within the SESN2 promoter, we generated five overlapping promoter fragments. The −229 to +200 bp region (Region 1) showed marked transcriptional activation by AGGF1 (Figure 4k).

### 2.5 AGGF1 Delays Endothelial Cell Senescence Through SESN2 Transcriptional Activation

**FIGURE 5:**
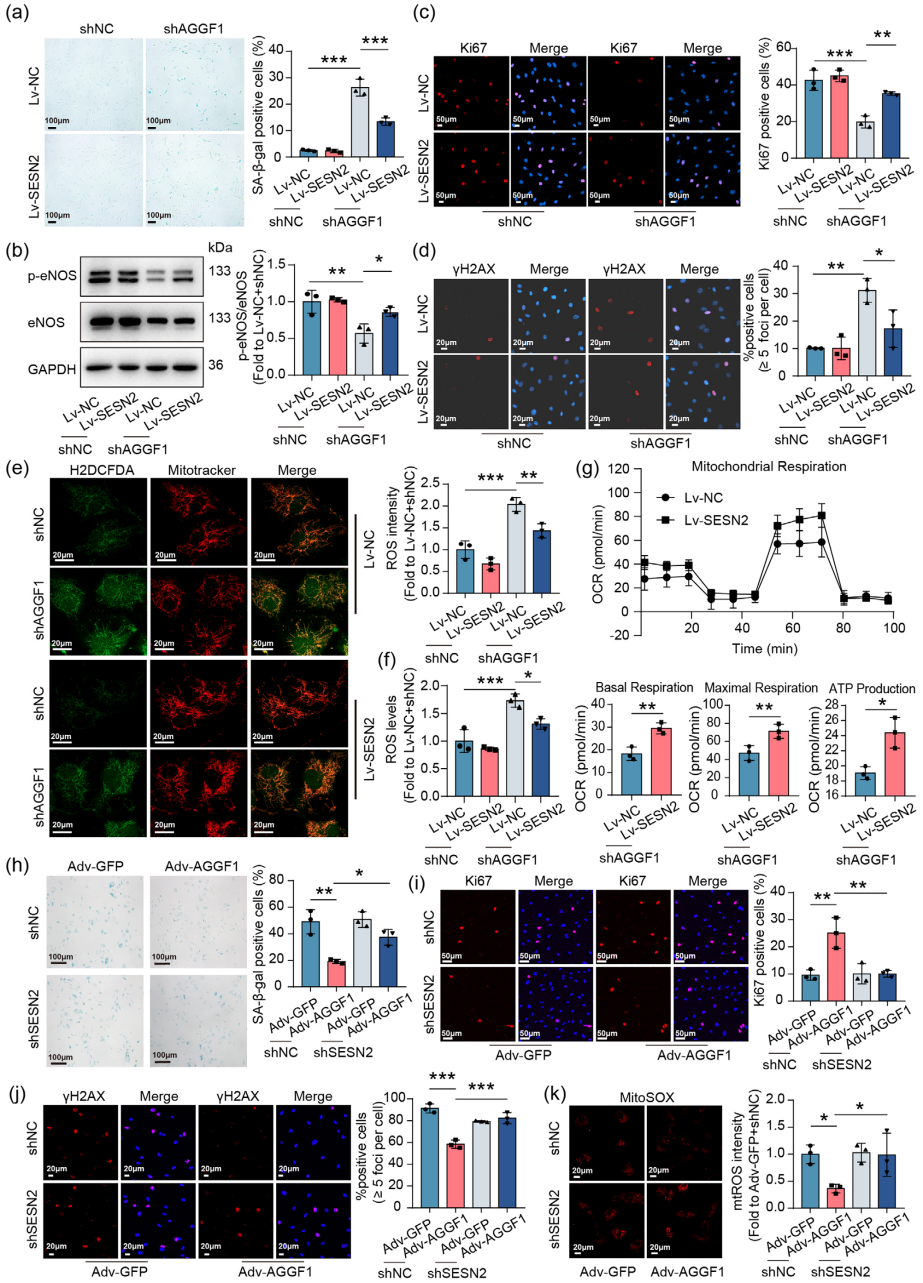
AGGF1 delays endothelial cell senescence through SESN2 transcriptional activation. HUVECs were infected with SESN2 lentivirus or control lentivirus for 24 h, followed by infection with shAGGF1 lentivirus or shNC lentivirus for another 24 h (a–g). Alternatively, HUVECs were infected with shSESN2 lentivirus or control lentivirus for 24 h, followed by infection with Adv‐AGGF1 adenovirus or control Adv‐GFP adenovirus for another 24 h, and then treated with DOX (h–k). (a, h) Representative images and quantification of SA‐β‐gal positive cells (*n* = 3 per group, 3 independent experiments), scale bar = 100 μm. One‐way ANOVA. (b) Western blot analysis of eNOS and p‐eNOS levels (*n* = 3 per group, 3 independent experiments). One‐way ANOVA. (c, i) Immunofluorescence staining for Ki67 (red) and Hoechst (blue) (*n* = 3 per group, 3 independent experiments), scale bar = 50 μm. One‐way ANOVA. (d, j) Immunofluorescence staining for γH2AX (red) and Hoechst (blue) (*n* = 3 per group, 3 independent experiments), scale bar = 20 μm. One‐way ANOVA. (e) Fluorescence staining of ROS (green, H_2_DCFDA) and mitochondria (red, MitoTracker) in HUVECs, with quantitative analysis of relative H_2_DCFDA fluorescence intensity (*n* = 3 per group, 3 independent experiments), scale bar = 20 μm. One‐way ANOVA. (f) ROS production was measured using a microplate reader with H_2_DCFDA (*n* = 3 per group, 3 independent experiments). One‐way ANOVA. (g) OCR was measured using the Seahorse XF Cell Mitochondrial Stress Test (*n* = 3 per group, 3 independent experiments). Two‐tailed Student's *t*‐test. (k) Representative images of mtROS detected by MitoSOX staining, with quantitative analysis of relative fluorescence intensity (*n* = 3 per group, 3 independent experiments), scale bar = 20 μm. One‐way ANOVA. Data are presented as mean ± SD. *p < 0.05, **p < 0.01, ***p < 0.001.

To investigate whether SESN2 is a key downstream effector molecule through which AGGF1 exerts its function, we performed gene overexpression and silencing experiments (Figure S10a,b). In AGGF1‐knockdown HUVECs, compared with Lv‐NC infection, Lv‐SESN2 infection significantly reduced the percentage of SA‐β‐gal positive cells (Figure 5a), increased p‐eNOS levels (Figure 5b), rescued the proportion of Ki67 positive cells (Figure 5c), and suppressed γH2AX levels (Figure 5d). Mitochondrial function assessment revealed that SESN2 overexpression enhanced maximal, basal, and ATP‐linked mitochondrial respiration (Figure 5g) and reduced mtROS generation (Figure 5e,f). Thus, SESN2 overexpression rescued AGGF1 deficiency‐induced senescence.

To further verify the relationship between AGGF1 and SESN2, we explored the impact of SESN2 knockdown on the regulatory effects of AGGF1 in DOX‐induced senescent HUVECs. In AGGF1‐overexpressing HUVECs, SESN2 knockdown increased the percentage of SA‐β‐gal positive cells (Figure 5h), decreased the proportion of Ki67 positive cells (Figure 5i), and elevated γH2AX levels (Figure 5j). SESN2 knockdown also increased mtROS (Figure 5k). Collectively, AGGF1 delays endothelial senescence through transcriptional activation of SESN2.

### 2.6 SESN2 Rescues Early Vascular Aging and Age‐Elevated Blood Pressure in *Aggf1^flox/flox^/Tie2‐Cre* ^+^ Mice

**FIGURE 6:**
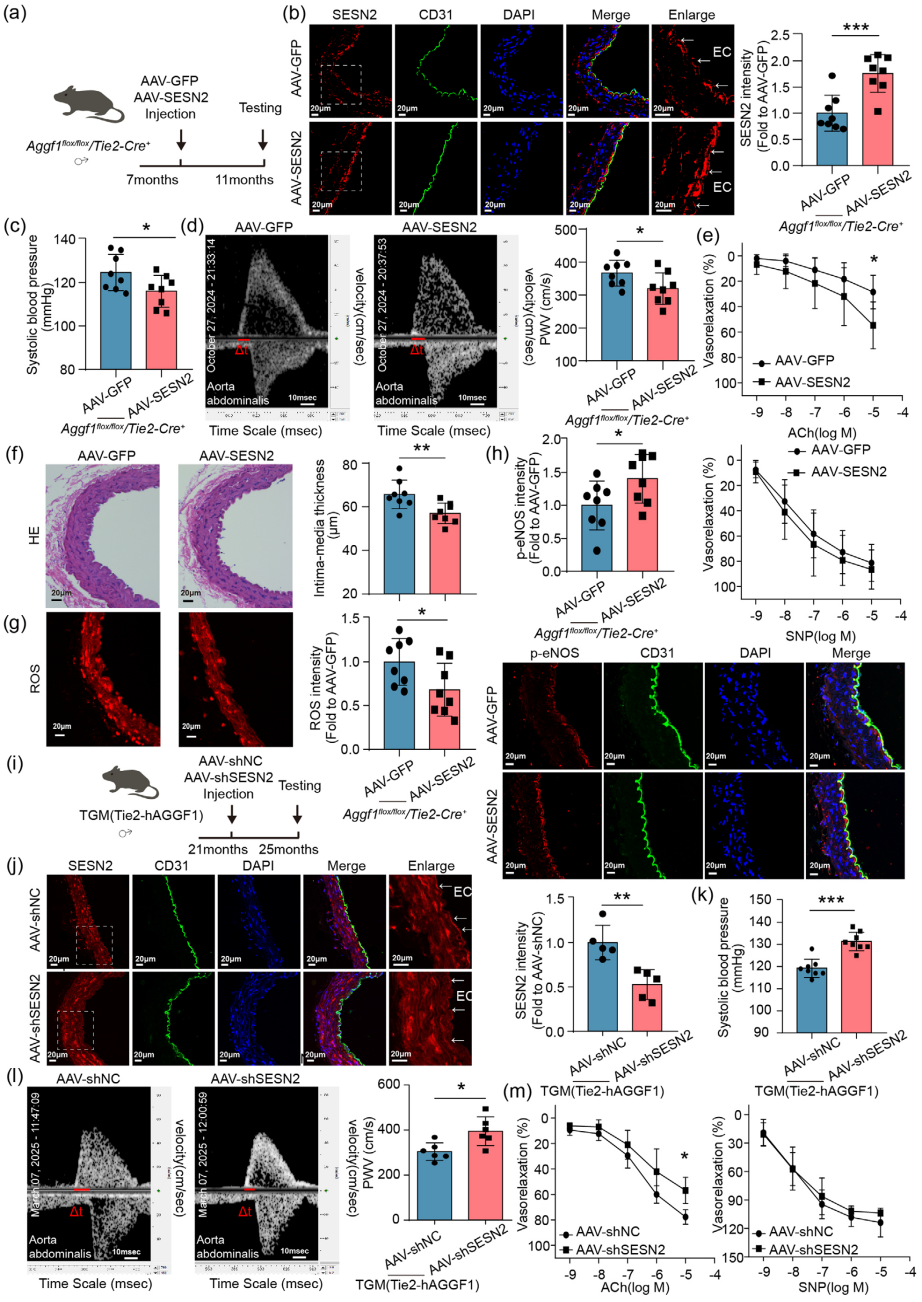
SESN2 rescues early vascular aging and age‐elevated blood pressure in *Aggf1* ^*flox/flox*^ */Tie2‐Cre* ^+^ mice. 7‐month‐old *Aggf1* ^*flox/flox*^ */Tie2‐Cre* ^+^ mice were injected intravenously with AAV‐SESN2 or AAV‐GFP (a–h). 21‐month‐old TGM(Tie2‐hAGGF1) mice were injected with AAV‐shSESN2 or AAV‐shNC (i–m). (a, i) Illustrated overview of the method used to generate endothelial‐specific SESN2 overexpression and knockdown mice. (b, j) Immunofluorescence staining for SESN2 in the vessels of *Aggf1* ^*flox/flox*^ */Tie2‐Cre* ^+^ mice (*n* = 8 per group) or TGM(Tie2‐hAGGF1) mice (*n* = 5 per group), scale bar = 20 μm. Two‐tailed Student's *t*‐test. (c, k) SBP measurement (*n* = 8 per group). Two‐tailed Student's *t*‐test. (d, l) Representative images and quantification of aortic PWV in *Aggf1* ^*flox/flox*^ */Tie2‐Cre* ^+^ mice (*n* = 8 per group) or TGM(Tie2‐hAGGF1) mice (*n* = 6 per group). Two‐tailed Student's *t*‐test. (e, m) Endothelium‐dependent and endothelium‐independent vasorelaxation were measured in aortic rings of *Aggf1* ^*flox/flox*^ */Tie2‐Cre* ^+^ mice (*n* = 8 per group) or TGM(Tie2‐hAGGF1) mice (*n* = 5 per group). Two‐way ANOVA. (f) Representative H&E staining and quantification of intima‐media thickness (*n* = 8 per group), scale bar = 20 μm. Two‐tailed Student's *t*‐test. (g) Fluorescence staining of ROS (red) in the aorta of *Aggf1* ^*flox/flox*^ */Tie2‐Cre* ^+^ mice (*n* = 8 per group), scale bar = 20 μm. Two‐tailed Student's *t*‐test. (h) Immunofluorescence staining for p‐eNOS (red), CD31 (green), and DAPI (blue) in aortas (*n* = 8 per group), scale bar = 20 μm. Two‐tailed Student's *t*‐test. Data are presented as mean ± SD. *p < 0.05, **p < 0.01, ***p < 0.001.

To investigate whether endothelial‐specific SESN2 overexpression could rescue early vascular aging and age‐elevated blood pressure in *Aggf1* ^*flox/flox*^ */Tie2‐Cre* ^+^ mice, a recombinant adeno‐associated virus (AAV) vector carrying the SESN2 gene under the control of the endothelial‐specific promoter (TIE) (AAV‐SESN2) was administered via tail‐vein injection (Figure 6a). Immunofluorescence analysis demonstrated elevated SESN2 levels in aortic endothelial cells of AAV‐SESN2‐treated mice (Figure 6b). Compared with AAV‐GFP controls, AAV‐SESN2‐treated mice showed reduced blood pressure (Figure 6c), decreased arterial stiffness (Figure 6d), improved endothelium‐dependent vasorelaxation (Figure 6e), and reduced aortic ROS levels with restored p‐eNOS (Figure 6g,h). Histological analysis using H&E staining revealed that AAV‐SESN2 treatment prevented aortic wall thickening (Figure 6f). Collectively, these data demonstrate that AGGF1 deficiency exacerbates early vascular aging and age‐elevated blood pressure through SESN2 downregulation.

Further investigation revealed that endothelial‐specific SESN2 knockdown attenuated the protective effects of AGGF1 on vascular aging and age‐elevated blood pressure. Immunofluorescence analysis demonstrated reduced SESN2 levels in aortic endothelial cells of AAV‐shSESN2‐treated mice (Figure 6i,j). TGM(Tie2‐hAGGF1) mice infected with AAV‐shSESN2 showed elevated blood pressure (Figure 6k), increased arterial stiffness (Figure 6l), and impaired endothelium‐dependent vasorelaxation (Figure 6m). Collectively, these findings establish the AGGF1/SESN2 signaling axis as a key protective mechanism against early vascular aging and age‐elevated blood pressure.

## 3 Discussion

In this study, we identify the endothelial AGGF1/SESN2/p‐eNOS axis as a novel and important signaling pathway in the maintenance of blood pressure. Age‐dependent attenuation of AGGF1 impairs SESN2 transcription, leading to mtROS accumulation, decreased p‐eNOS, and endothelial cell dysfunction. This signaling cascade is critical for maintaining mitochondrial function, and its suppression promotes early vascular aging and elevates blood pressure.

Aging is an important independent risk factor for both prehypertension and hypertension (Kong et al. 2020; Wang and Wang 2004). With advancing age, endothelial cells exhibit reduced NO production, a key mediator of vascular relaxation, leading to impaired blood pressure regulation (Long et al. 2022; Młynarska et al. 2025; Smith et al. 2020; Sun 2015). One study indicated AGGF1 levels were significantly decreased in hypertensive patients (Gao et al. 2026), suggesting that reduced AGGF1 levels might be associated with elevated blood pressure. Nevertheless, direct evidence that AGGF1 regulates blood pressure is lacking. In this study, we found that AGGF1 expression was downregulated in the arterial tissues of aged mice with elevated blood pressure, suggesting its role in the interplay between aging and blood pressure regulation. Importantly, we confirmed that endothelial‐specific knockout of AGGF1 led to age‐elevated blood pressure in mice, whereas endothelial‐specific overexpression of AGGF1 effectively lowered blood pressure in aged mice. Notably, the systolic blood pressure of endothelial‐specific AGGF1 knockout mice did not exceed 139 mmHg, indicating that AGGF1 deficiency contributes to the development of prehypertension.

Early vascular aging represents a critical pathological link between aging and age‐elevated blood pressure (Laurent et al. 2019; Long et al. 2022). Endothelial dysfunction accelerates the progression of vascular aging by triggering oxidative stress, reducing NO bioavailability, and promoting the release of pro‐inflammatory cytokines, all of which contribute to elevated levels of ROS in vascular smooth muscle cells (Ledet et al. 2014; Schiavi et al. 2018; Xu et al. 2009). However, the role of AGGF1 in early vascular aging remains uncharacterized. Here, we demonstrated that endothelial AGGF1 deficiency induced multiple hallmarks of vascular aging, including increased arterial stiffness, intima‐media thickening, augmented collagen deposition, impaired endothelium‐dependent vasodilation, increased ROS production, and decreased eNOS phosphorylation, whereas AGGF1 overexpression in endothelial cells rescued these phenotypic changes. Endothelial premature senescence is a critical driver of early vascular aging (Tian and Li 2014). In vitro, AGGF1 knockdown in HUVECs induced senescence, whereas AGGF1 overexpression rescued endothelial cell senescence. Collectively, these findings provide compelling evidence for a novel function of AGGF1 in regulating early vascular aging. Vascular aging is a key driver of systemic aging and an important risk factor for shortened lifespan (Grunewald et al. 2021; Pietri and Stefanadis 2021). Our findings suggest that AGGF1 may modulate systemic aging, a possibility that merits further investigation.

The precise regulation of blood pressure relies on the synergistic interactions of multiple cells and mechanisms. Endothelial cells modulate vasodilation by secreting vasoactive substances such as NO, whereas vascular smooth muscle cells directly control vascular tone through contraction and relaxation (J. Ma et al. 2023). Both our previous and present data demonstrate that during aging, endothelium‐dependent vasodilation is significantly impaired, whereas smooth muscle‐dependent vasodilation remains unchanged (Long et al. 2022; Zhao, Qiu, et al. 2025). This suggests that endothelial cells, rather than smooth muscle cells, play a central role in age‐elevated blood pressure, which underpins our investigation into the role of AGGF1 in endothelial cells. Notably, our findings indicate that endothelial AGGF1 deficiency elevates blood pressure in aged mice but not in young mice. With advancing age, progressive endothelial dysfunction, cumulative mitochondrial oxidative stress, and increased arterial stiffness collectively constitute the pathological basis for age‐elevated blood pressure (Harvey et al. 2015; Sun 2015). Our data demonstrate that AGGF1 plays a crucial vasoprotective role in aging by mitigating oxidative stress and preserving endothelial homeostasis. Consequently, AGGF1 deficiency in aged mice eliminates this protective mechanism, resulting in age‐elevated blood pressure.

Mitochondrial dysfunction is a critical factor in the development of elevated blood pressure (Forte et al. 2020; Griendling et al. 2021). Our previous research identified mitochondrial dysfunction as a key driver of vascular aging, which impairs endothelial function through mechanisms such as excessive ROS production (Yu et al. 2024). We found that AGGF1 knockdown impaired mitochondrial respiration and increased ROS production, whereas AGGF1 overexpression alleviated these effects, underscoring the central role of AGGF1 in regulating endothelial mitochondrial function. Further investigation demonstrated that SESN2 overexpression rescued the mitochondrial respiratory dysfunction, ROS accumulation, and reduction in p‐eNOS induced by AGGF1 knockdown. ROS have been demonstrated to inhibit eNOS phosphorylation (He et al. 2019; Lamoke et al. 2015; Wang et al. 2019). Therefore, AGGF1 improves mitochondrial function through SESN2, thereby promoting eNOS phosphorylation.

Another key finding of this study is that SESN2 is a direct downstream molecule of AGGF1 in regulating age‐elevated blood pressure. As a conserved stress‐responsive protein, SESN2 plays an important role in maintaining cellular homeostasis and regulating vascular function (Fatima et al. 2021; Lu et al. 2023). We demonstrated that AGGF1 directly bound to the SESN2 promoter and enhanced its transcription. Crucially, SESN2 overexpression rescued AGGF1 deficiency‐induced elevated blood pressure, whereas SESN2 knockdown attenuated the beneficial vascular effects of AGGF1, confirming that SESN2 acts as an essential downstream effector. Although renal SESN2 has been implicated in blood pressure regulation (Yang, Cuevas, et al. 2014), our study is the first to demonstrate a specific role of endothelial SESN2 in regulating age‐elevated blood pressure. However, SESN2 overexpression partially rescued the defects induced by AGGF1 deficiency, indicating that additional mediators cooperate with the SESN2 pathway to mediate AGGF1‐dependent vascular protection. Additionally, since the AGGF1/SESN2 axis was defined in endothelial cells, its conservation in other vascular cell types requires further investigation.

This study has some limitations. First, although we demonstrated that reduced AGGF1 elevates blood pressure in the mouse model and also observed AGGF1 downregulation in both the peripheral blood of hypertensive patients and the vasculature of elderly individuals, suggesting its potential role in the regulation of age‐elevated blood pressure in human populations, more direct evidence is needed to confirm this association. Second, the reason for the decrease in AGGF1 expression during aging has not been elucidated. AGGF1 expression is precisely regulated by multiple transcription factors. Notably, the expression of transcription factors such as HIF‐1α and BACH1 is altered during aging (Alique et al. 2020; Ge et al. 2021). These age‐related changes in transcription factors may contribute to reduced AGGF1 expression during aging, providing important clues for future investigations into the upstream regulatory mechanisms of AGGF1. Moreover, this study was conducted exclusively in male mice, and our findings are applicable only to males. Given substantial evidence indicating significant sex differences in hypertension (Gillis and Sullivan 2016), future studies should investigate the role of AGGF1 in blood pressure regulation in females.

In this study, blood pressure was measured at discrete time points using both carotid artery cannulation and tail‐cuff methods. Although the reliability of these two methods has been validated by telemetry in other studies (Chen and Sun 2018; Feng et al. 2008; Whitesall et al. 2004), the lack of continuous telemetric monitoring in our experiments precluded identification of the exact age at which blood pressure began to increase. Additionally, the H_2_DCFDA probe used for ROS detection does not directly react with oxidants and may be prone to artifacts (Kalyanaraman et al. 2012). However, the consistency with the MitoSOX Red staining results supports our conclusions regarding AGGF1‐mediated oxidative stress regulation. Furthermore, although the Tie2‐Cre transgenic mouse is a well‐established model for endothelial‐targeted gene deletion, the Tie2 (TEK) gene itself is expressed at low levels in several other cell types, such as glial cells and cells of the hematopoietic lineage. Therefore, we cannot completely exclude the influence of these cells on blood pressure regulation.

In summary, our study is the first to establish that the endothelial AGGF1/SESN2/p‐eNOS signaling axis plays a pivotal role in blood pressure regulation in male mice. Aging attenuates the AGGF1/SESN2/p‐eNOS signaling cascade, thereby promoting early vascular aging and elevating blood pressure. These results provide novel insights into age‐elevated blood pressure in male mice and suggest AGGF1 as a potentially valuable target for prehypertension intervention in the elderly male population.

## 4 Experimental Procedures

### 4.1 Animal Models

*Aggf1* ^*flox/flox*^ mice and Tie2‐Cre mice were obtained from Gempharmatech Corporation. *Aggf1* ^*flox/flox*^ mice were crossed with mice expressing Cre recombinase under the control of the Tie2 promoter (Tie2‐Cre) to generate endothelial‐specific *Aggf1* knockout mice (*Aggf1* ^*flox/flox*^ */Tie2‐Cre* ^+^). Genomic DNA extracted from mouse tails was genotyped via polymerase chain reaction (PCR) using specific primers. The floxed gene genotyping primers (forward: 5′‐CGTGATAGTAAGGCTTTGAGGG‐3′; reverse: 5′‐GGTCAATAGAGCCAACGATAAG‐3′) produced 467‐bp and 349‐bp fragments for mutant and wild‐type mice, respectively. Cre gene genotyping primers (forward: 5′‐GCCTGCATTACCGGTCGATGC‐3′; reverse: 5′‐CTAAGTGCCTTCTCTACACCTGC‐3′) produced a 609‐bp fragment in Tie2‐Cre mice, while wild‐type mice showed no amplification. Endothelial‐specific *AGGF1* overexpression mice (TGM(Tie2‐hAGGF1)) were obtained from the Animal Center of Peking University. To generate TGM(Tie2‐hAGGF1) mice, the human *AGGF1* coding sequence was cloned into the pSPTg.T2FpAXK vector. This vector incorporated the endothelium‐specific Tie2 promoter. Potential transgenic founders were identified by PCR of genomic DNA extracted from tail tips, using primers specific for human *AGGF1* (forward, 5′‐TGCTGCATCACACAGAACGG‐3′; reverse, 5′‐ACTTTCAGCTAATGATGAGCCTT‐3′), producing a 434‐bp PCR product. Male C57BL/6 mice were purchased from Gempharmatech Corporation.

### 4.2 Animal Experiments

Only male mice were used to eliminate potential estrogen‐mediated sex differences. Mice were housed in specific pathogen‐free (SPF) cages with free access to water and food, and were maintained under a strict 12‐h light/dark cycle in an SPF environment. All animal experiments were approved by the Laboratory Animal Ethics Committee of Nanchang University (Approval No. NCULAE‐20221130018) and were conducted in accordance with the National Institutes of Health guidelines. All mice were anesthetized via a single intraperitoneal injection of pentobarbital sodium (50 mg/kg). Euthanasia was performed by a single intraperitoneal injection of pentobarbital sodium (150 mg/kg).

Mice were randomly assigned to experimental groups, with investigators blinded to group allocation during procedures and assessments. Specifically, for image analysis and data quantification, all images were anonymized with unique coding by a third‐party researcher prior to analysis, with no group identifiers in file names, metadata, or image content. Quantification was independently performed by two researchers, both blinded to group allocation. For each image, the following parameters were quantified using ImageJ software: intima‐media thickness, collagen deposition area, ROS fluorescence intensity, and the immunofluorescence intensities of AGGF1, p‐eNOS, and SESN2. Measurements from the two researchers were averaged for each image. Group information was disclosed by the third‐party researcher holding the coding key only after all data had been collected, quantified, and recorded. Data were then merged with group labels and statistically analyzed using GraphPad Prism 8.0. Results were interpreted in the context of experimental groups only after analysis was completed.

### 4.3 Cell Culture and Treatments

HUVECs were obtained from the Cell Bank of the Chinese Academy of Sciences and cultured in endothelial cell medium (ECM, ScienCell). HEK293A and HEK293T cells were obtained from ATCC (Manassas, VA, USA) and cultured in Dulbecco's Modified Eagle Medium (DMEM, Corning) supplemented with 10% fetal bovine serum (FBS, Clark). All cells were maintained at 37°C in 5% CO_2_ and 95% air. Cells were treated with doxorubicin (DOX, 25 nM, BMASSAY, #C6557) for 24 h.

### 4.4 Plasmid Construction

Recombinant adenoviruses were generated using the AdEasy system (Stratagene) as described in our previous report (Hu et al. 2013). Full‐length human *AGGF1* was subcloned from pcDNA3.1‐AGGF1 into pShuttle‐IRES‐hrGFP‐1 with a Flag tag at the C‐terminus. The AGGF1‐ΔNLS mutant, in which the putative nuclear localization signal (NLS) region (amino acids 260–288) was deleted, was generated as previously described (Zhang et al. 2021). The lentiviral vector for SESN2 overexpression was constructed by cloning the *SESN2* coding sequence (CDS) into the pLenti vector. shRNAs targeting *AGGF1* or *SESN2* were subcloned into the pLKO.1 plasmid under the U6 promoter. The target sequences for *AGGF1* were 5′‐GGAGAAGTTGGAACGTGAACT‐3′ and 5′‐GATAGTTATGACGAAGCCATT‐3′. The target sequence for *SESN2* was 5′‐GAAGACCCTACTTTCGGATAT‐3′. All constructs were verified by DNA sequencing (Tsingke). The vectors were co‐transfected with pMD2.G and psPAX2 into HEK293T cells for packaging. Viral supernatants were harvested at 48 and 72 h after transfection and filtered through 0.45‐μm membranes (Merck).

### 4.5 Blood Pressure Measurements

Blood pressure of conscious mice was measured using a noninvasive tail‐cuff system (IITC Life Sciences) between 15:00 and 17:00. Mice were acclimatized to restraint and tail‐cuff procedures for 7 days prior to measurements. Five consecutive pulse readings were recorded for each mouse, with each inflation/deflation cycle lasting 10–15 s.

For direct measurement of carotid artery blood pressure, mice were anesthetized by a single intraperitoneal injection of pentobarbital sodium (50 mg/kg). Following tracheostomy, a polyethylene catheter was inserted into the right carotid artery. Blood pressure was measured using a pressure transducer (Ohmeda) connected to a blood pressure analyzer (Micromed), as previously described.

### 4.6 Vascular Function Studies

Thoracic aortas were excised, cleaned of perivascular adipose tissue, and sectioned into 2–3 mm rings. Aortic rings were mounted in pressure myographs (Danish Myo Technology) for endothelial function assessment. Rings were maintained in modified Krebs–Henseleit solution (118 mmol·L^−1^ NaCl, 4.7 mmol·L^−1^ KCl, 2.5 mmol·L^−1^ CaCl_2_, 1.2 mmol·L^−1^ MgSO₄, 1.2 mmol·L^−1^ KH_2_PO₄, 25 mmol·L^−1^ NaHCO₃, and 11 mmol·L^−1^ glucose) and equilibrated at 4 mN intraluminal pressure for 60 min under 95% air and 5% CO_2_. After pre‐contraction with phenylephrine (1 μmol·L^−1^), endothelium‐dependent and ‐independent vasodilation were evaluated using acetylcholine (ACh, 10^−9^–10^−5^ mol·L^−1^) and sodium nitroprusside (SNP, 10^−9^–10^−5^ mol·L^−1^) responses. Concentration‐response curves to ACh and SNP were analyzed.

### 4.7 Senescence‐Associated β‐Galactosidase Staining

SA‐β‐gal staining was performed using a senescence detection kit (Beyotime) according to the manufacturer's instructions. HUVECs were washed with PBS, fixed in 4% paraformaldehyde for 15 min, and incubated with β‐galactosidase staining solution at 37°C for 12 h. SA‐β‐gal positive cells (blue staining) were quantified by counting 10 random fields per sample under light microscopy.

### 4.8 Histological Analysis of Tissues

Aortic sections were prepared from the distal descending aorta (after the origin of the left subclavian artery from the aortic arch). The thoracic aorta was fixed in 4% paraformaldehyde (PFA) for 24 h, embedded in paraffin, and sectioned at 5 μm thickness. Masson's trichrome and hematoxylin–eosin (H&E) staining were performed using commercial kits (Solarbio). H&E staining evaluated vascular morphology, while Masson's trichrome staining assessed collagen deposition. Images were acquired using light microscopy, and intima‐media thickness as well as collagen deposition was quantified using ImageJ.

### 4.9 Reactive Oxygen Species Measurement

ROS production was measured using a microplate reader with 2′,7′‐dichlorodihydrofluorescein diacetate (H_2_DCFDA, to assess intracellular total ROS). Cells in 96‐well plates were incubated with 10 μmol·L^−1^ H_2_DCFDA (Thermo Fisher) for 30 min at 37°C. Fluorescence was measured at 488 nm excitation and 525 nm emission using a microplate reader (BioTek).

For vital imaging of oxidative stress, ROS production was measured using confocal microscopy with H_2_DCFDA or MitoSOX Red (to detect mtROS). Cells in glass‐bottom dishes were co‐incubated with 10 μmol·L^−1^ H_2_DCFDA and 100 nmol·L^−1^ MitoTracker Deep Red FM (Thermo Fisher) for 30 min. Cells were incubated with 1 μmol·L^−1^ MitoSOX Red (Thermo Fisher) for 30 min. Aortic cryosections (5 μm) were stained using an ROS assay kit (Sigma, MAK142) for 30 min. Fluorescence images were captured using a confocal microscope (LSM 800, Zeiss) with standardized imaging parameters.

### 4.10 Western Blot Analysis

Protein extraction and immunoblotting were performed according to standard protocols. Cells or tissues were homogenized in RIPA buffer supplemented with protease and phosphatase inhibitors. Following centrifugation (12,000×*g*, 15 min, 4°C), protein concentrations were measured using a bicinchoninic acid (BCA) assay kit. Equal amounts of protein were separated by sodium dodecyl sulfate‐polyacrylamide gel electrophoresis (SDS‐PAGE), transferred to polyvinylidene fluoride (PVDF) membranes (Millipore), and incubated with primary antibodies. Protein bands were detected using an enhanced chemiluminescence (ECL) substrate (Sailin Biotechnology) and quantified using ImageJ, with normalization to GAPDH or β‐actin. Detailed antibody information is provided in Table S1.

### 4.11 Immunofluorescence

Cells in 12‐well plates were fixed with ice‐cold methanol, blocked with 1% BSA, and incubated with primary antibodies at 4°C overnight. After washing with PBST, cells were incubated with fluorescent secondary antibodies and Hoechst or 4′,6‐diamidino‐2‐phenylindole (DAPI), and then mounted in glycerol‐gelatin for confocal microscopy (LSM 800, Zeiss). For aortic immunofluorescence, frozen sections were blocked with 5% BSA, incubated with primary antibodies overnight, and subsequently stained with secondary antibodies for 1 h. Nuclei were counterstained with Hoechst or DAPI. Sections were imaged using the same confocal microscope settings.

### 4.12 RNA Extraction and qRT‐PCR

Total RNA was extracted from cells using TRIzol (BMASSAY), and complementary DNA (cDNA) was synthesized using a reverse transcription kit (Promega). qRT‐PCR was performed using a 7300 Real‐Time PCR System (Applied Biosystems) with SYBR Green Master Mix (Yeasen), with normalization to GAPDH. Primer sequences are listed in Table S2.

### 4.13 RNA Sequencing Analysis

Cultured HUVECs were harvested 48 h after AGGF1 overexpression or knockdown, and total RNA was extracted using the TRIzol method. RNA libraries were prepared for paired‐end RNA sequencing on the DNBSEQ‐T7 platform (Oebiotech, Shanghai, China). FASTQ data were aligned to the human genome using HISAT2 software. Differential expression analyses were performed using DESeq2 software. |Fold change| > 1.5 and *q*‐values < 0.05 were set as the thresholds for differential expression. Enrichment analyses, including Gene Ontology and pathway analyses, were performed using R (v 4.4.1).

### 4.14 Luciferase Assays

The human SESN2 promoter (−1610 to +200 bp) and its truncated fragments (R1: −229 to +200 bp; R2: −588 to −171 bp; R3: −923 to −517 bp; R4: −1273 to −874 bp; R5: −1610 to −1166 bp) were cloned into the pGL6‐TA luciferase vector. HEK293T cells were co‐transfected with each pGL6‐TA reporter construct, the pRL‐TK plasmid, and either the AGGF1 expression plasmid or the empty vector. After 48 h of treatment, the cells were lysed. Firefly luciferase activity was measured as previously described (Yang, Zhou, et al. 2014) and normalized to Renilla luciferase activity.

### 4.15 Adeno‐Associated Virus Delivery

Adeno‐associated viruses under the control of the endothelial‐specific promoter (TIE) and with an AAV^ENT^ serotype were packaged by Shanghai GeneChem to induce endothelium‐specific knockdown or overexpression of SESN2 in mice (Ren et al. 2023). The AAV containing a short hairpin RNA targeting the SESN2‐Mus sequence (AAV^ENT^‐TIE‐SESN2 shRNA, AAV‐shSESN2) or a scrambled sequence (AAV^ENT^‐TIE‐NC shRNA, AAV‐shNC) was constructed with a titer of 1 × 10^13^ vg·mL^−1^. The target sequence for SESN2 was 5′‐TATGATTACGGCGAGGTAAAC‐3′. The AAV containing the full‐length SESN2 TIEp‐SESN2‐EGFP‐MCS‐SV40 PolyA (AAV‐SESN2) or the corresponding control vector TIEp‐EGFP‐MCS‐SV40 PolyA (AAV‐GFP) was constructed with a titer of 1 × 10^13^ vg·mL^−1^.

In 21‐month‐old male TGM(Tie2‐hAGGF1) mice, AAV‐shSESN2 or AAV‐shNC was administered via tail‐vein injection at a dose of 3 × 10^11^ vg per mouse in 200 μL saline. Similarly, AAV‐SESN2 or AAV‐GFP was injected into 7‐month‐old male *Aggf1* ^*flox/flox*^ */Tie2‐Cre* ^+^ mice at the same dose. No apparent toxicity was observed in preliminary studies.

### 4.16 Seahorse Metabolic Analysis

The oxygen consumption rate was measured in HUVECs seeded at 2 × 10^4^ cells per well in XF24 plates using the Seahorse XF Cell Mitochondrial Stress Test Kit (Agilent Technologies). After incubation in XF base medium, oligomycin (1 μmol·L^−1^), carbonyl cyanide 4‐(trifluoromethoxy)phenylhydrazone (FCCP, 2 μmol·L^−1^), and rotenone/antimycin A (0.5 μmol·L^−1^) were added sequentially. Data were analyzed using Seahorse Wave software.

### 4.17 NO Measurement

NO production was determined by measuring nitrite (NO_2_^−^) accumulation in culture supernatants using a Griess assay kit (Beyotime) according to the manufacturer's protocol. HUVECs were seeded and subjected to the indicated treatments. Conditioned medium was collected and centrifuged to remove cell debris. Equal volumes of supernatant (50 μL) were mixed with 50 μL of Griess reagent I and 50 μL of Griess reagent II in a 96‐well plate, and absorbance was measured at 540 nm using a microplate reader (BioTek). Nitrite concentrations were calculated from a standard curve generated with sodium nitrite (NaNO_2_).

### 4.18 Pulse Wave Velocity

Anesthetized mice were placed in the supine position on 37°C heating pads. Pulse waves were detected at the thoracic aortic arch and abdominal aorta using a Doppler probe (Indus Instruments). PWV was calculated as (probe distance)/(abdominal time—thoracic time).

### 4.19 Statistical Analysis

Data are presented as mean ± standard deviation (SD) and analyzed using GraphPad Prism 8.0. Two‐tailed Student's *t*‐test was performed for comparisons between two groups. For single‐variable data among three or more groups, one‐way ANOVA followed by Tukey's post hoc test was performed. For comparisons involving multiple groups with two or more variables, two‐way ANOVA followed by Tukey's post hoc test was used. *p < 0.05, **p < 0.01, and ***p < 0.001 were considered statistically significant.

## Author Contributions

Xiao‐Li Tian conceptualized and supported the study. Xiao‐Li Tian, Weixin Lv, and Xiaojuan Zhong designed the study. Weixin Lv and Xiaojuan Zhong performed experiments and conducted data analysis. Qiang Yuan and Shilin Zhang assisted with experimental procedures. Xueting Gong, Ya Zhao, Andong Wu, Ming Wan, Xueer Li, Yangyi Zheng, Jiankun Liu, Bingbing Zhou, Yuanzheng Zhu, Limin Zhao, Yang Xiang, and Qiquan Wang contributed to scientific discussions and intellectual input. Andong Wu, Yang Xiang, and Qiquan Wang performed the English editing of the manuscript. Weixin Lv prepared the manuscript. Xiao‐Li Tian conducted revision and editing. All authors read and approved the final manuscript.

## Funding

This work was supported by grants from the Key Program of the National Natural Science Foundation of China (82330046) and the National Key Research and Development Program of the Ministry of Science and Technology, China (2023YFC3603300), and the Jiangxi Province Key Laboratory of Aging and Disease (2024SSY07161).

## Disclosure

Permission Statement: All data generated in this study are available.

## Conflicts of Interest

The authors declare no conflicts of interest.

## Supporting information


**Figure S1:** Generation and genotyping of *Aggf1* ^*flox/flox*^ */Tie2‐Cre* ^+^ mice and TGM(Tie2‐hAGGF1) mice. (a) General strategy for the generation of *Aggf1* ^*flox/flox*^ */Tie2‐Cre* ^+^ mice. *Aggf1* ^*flox/flox*^ mice were crossed with Tie2‐Cre mice to generate endothelial‐specific *Aggf1* knockout mice (*Aggf1* ^*flox/flox*^ */Tie2‐Cre* ^+^ mice). Two loxP sites flank exon 3 of the *Aggf1* gene. Exon 3 is removed when the Tie2 promoter drives the expression of the Cre recombinase. (b) General strategy for the generation of TGM(Tie2‐hAGGF1) mice. Genotyping was confirmed by tail biopsy and PCR at 2 weeks of age.

**Figure S2:** Endothelium‐dependent and endothelium‐independent vasorelaxation in aged mice. (a) Endothelium‐dependent vasorelaxation responses to ACh were measured in aortic rings (*n* = 6 per group). Two‐way ANOVA. (b) Endothelium‐independent vasorelaxation responses to SNP were measured in aortic rings (*n* = 6 per group). Two‐way ANOVA. Data are presented as mean ± SD. *p < 0.05, **p < 0.01, ***p < 0.001.

**Figure S3:** Validation of AGGF1 overexpression and knockdown in HUVECs. (a) AGGF1 mRNA expression was markedly decreased in HUVECs infected with shAGGF1 lentivirus (*n* = 3 per group, 3 independent experiments). One‐way ANOVA. (b) AGGF1 mRNA expression was significantly increased in HUVECs infected with Adv‐AGGF1 adenovirus (*n* = 3 per group, 3 independent experiments). Two‐tailed Student's *t*‐test. (c) AGGF1 mRNA expression was significantly increased in HUVECs infected with Lv‐AGGF1 lentivirus (*n* = 3 per group, 3 independent experiments). Two‐tailed Student's *t*‐test. Data are presented as mean ± SD. ***p < 0.001.

**Figure S4:** AGGF1 knockdown reduces NO production and upregulates IL‐6 expression in HUVECs. (a) Quantification of NO concentration in the culture supernatant of HUVECs (*n* = 3 per group, 3 independent experiments). One‐way ANOVA. (b) IL‐6 mRNA expression was analyzed by qRT‐PCR (*n* = 3 per group, 3 independent experiments). One‐way ANOVA. Data are presented as mean ± SD. *p < 0.05, **p < 0.01.

**Figure S5:** DOX induces endothelial cell senescence. HUVECs were treated with 25 nM DOX for 24 h. (a) Representative images and quantification of SA‐β‐gal positive cells (*n* = 3 per group, 3 independent experiments), scale bar = 100 μm. Two‐tailed Student's *t*‐test. (b) Western blot analysis of eNOS and p‐eNOS (*n* = 3 per group, 3 independent experiments). Two‐tailed Student's *t*‐test. (c) Immunofluorescence staining for γH2AX (red) and Hoechst (blue) in HUVECs (*n* = 3 per group, 3 independent experiments), scale bar = 20 μm. Two‐tailed Student's *t*‐test. (d) Immunofluorescence staining for Ki67 (red) and Hoechst (blue) in HUVECs (*n* = 3 per group, 3 independent experiments), scale bar = 20 μm. Two‐tailed Student's *t*‐test. (e) Representative images of mtROS detected by MitoSOX staining, with quantitative analysis of relative fluorescence intensity (*n* = 3 per group, 3 independent experiments), scale bar = 20 μm. Two‐tailed Student's *t*‐test. Data are presented as mean ± SD. *p < 0.05, ***p < 0.001.

**Figure S6:** Heatmap of mitochondrial pathway‐related genes co‐expressed with AGGF1. Among the 199 differentially co‐expressed genes, mitochondrial pathway‐related genes were identified by GO enrichment analysis.

**Figure S7:** SESN2 expression is positively correlated with AGGF1 in human vasculature and declines with age in mouse aortic endothelium. (a) Correlation analysis of SESN2 and AGGF1 expression in the human vascular tissues of elderly individuals from the ADEIP database. Spearman correlation analysis revealed a significant positive correlation (*r* = 0.2807, *p* < 0.001). (b) Immunofluorescence staining for SESN2 (red), CD31 (green), and DAPI (blue) in the aortic endothelium of young (4‐month‐old), middle‐aged (11‐month‐old), and aged (25‐month‐old) mice (*n* = 6 per group), white scale bar = 20 μm, blue scale bar = 10 μm. One‐way ANOVA. Data are presented as mean ± SD. *p < 0.05, ***p < 0.001.

**Figure S8:** AGGF1 exhibits nuclear localization in quiescent endothelial cells. Immunofluorescence staining for AGGF1 (green) and Hoechst (blue) in HUVECs, scale bar = 5 μm.

**Figure S9:** Nuclear localization of AGGF1 is required for its protective effect against endothelial cell senescence. (a) Immunofluorescence staining for AGGF1 (green) and Hoechst (blue). The ΔNLS mutant protein was absent from the nucleus, scale bar = 20 μm. (b) Representative images and quantification of SA‐β‐gal positive cells (*n* = 3 per group, 3 independent experiments), scale bar = 100 μm. Two‐tailed Student's *t*‐test. Data are presented as mean ± SD. ns, not significant.

**Figure S10:** Validation of SESN2 overexpression and knockdown in HUVECs. (a) SESN2 expression was significantly increased in HUVECs infected with SESN2 lentivirus (*n* = 3 per group, 3 independent experiments). Two‐tailed Student's *t*‐test. (b) SESN2 mRNA expression was significantly decreased in HUVECs infected with shSESN2 lentivirus (*n* = 3 per group, 3 independent experiments). Two‐tailed Student's *t*‐test. Data are presented as mean ± SD. ***p < 0.001.

**Table S1:** Antibodies.
**Table S2:** Primers.

## Data Availability

The data that support the findings of this study are openly available in NCBI SRA at https://www.ncbi.nlm.nih.gov/bioproject/PRJNA1493849/, reference number PRJNA1493849.
