# Supplementary figures and images for: Endothelial AGGF1 Deficiency Causes Mitochondrial Dysfunction and Contributes to Age‐Elevated Blood Pressure
Source: Aging Cell. 2026 Aug 3;25(8):e70652. doi: 10.1111/acel.70652 (PMC13430426; doi:10.1111/acel.70652)

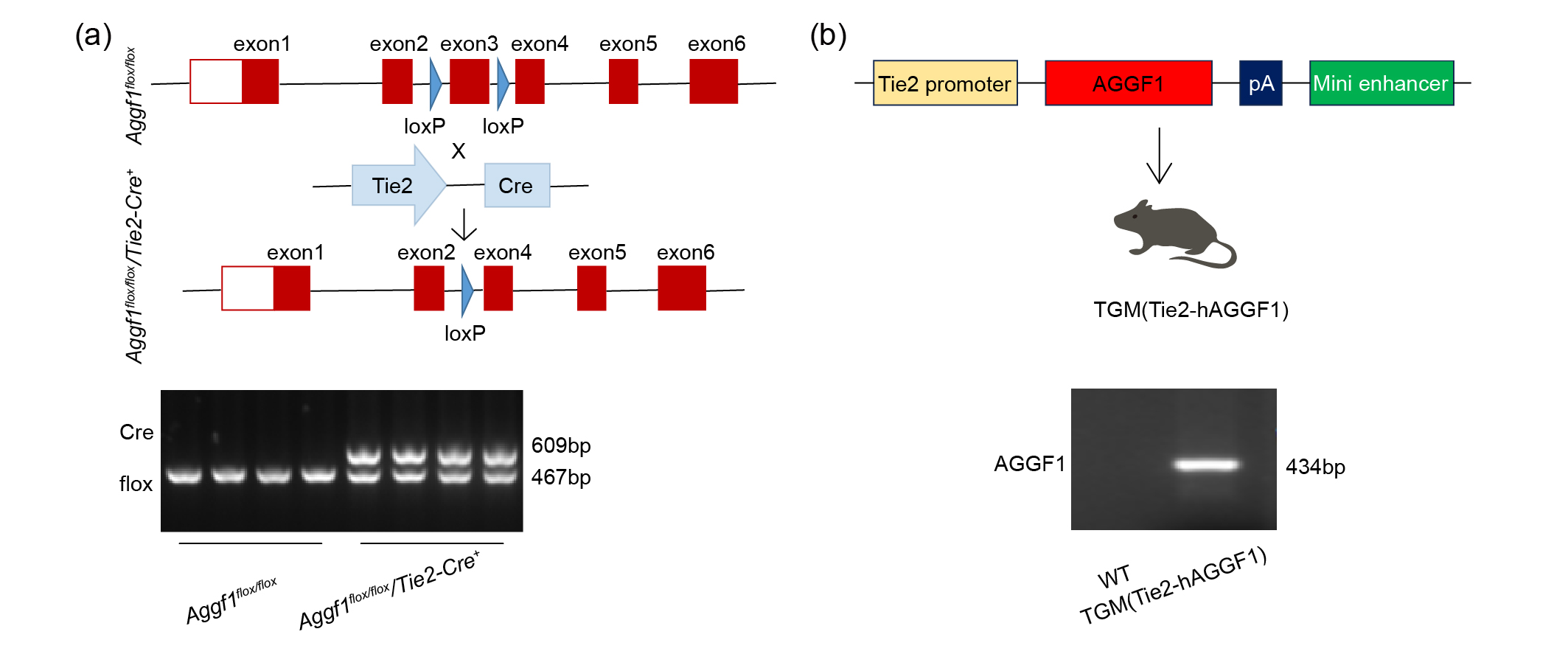

Supplement: Supplementary file 1 — Figure S1: Generation and genotyping of Aggf1 flox/flox /Tie2‐Cre + mice and TGM(Tie2‐hAGGF1) mice. (a) General strategy for the generation of Aggf1 flox/flox /Tie2‐Cre + mice. Aggf1 flox/flox mice were crossed with Tie2‐Cre mice to generate endothelial‐specific Aggf1 knockout mice (Aggf1 flox/flox /Tie2‐Cre + mice). Two loxP sites flank exon 3 of the Aggf1 gene. Exon 3 is removed when the Tie2 promoter drives the expression of the Cre recombinase. (b) General strategy for the generation of TGM(Tie2‐hAGGF1) mice. Genotyping was confirmed by tail biopsy and PCR at 2 weeks of age. [file ACEL-25-e70652-s007.jpg]

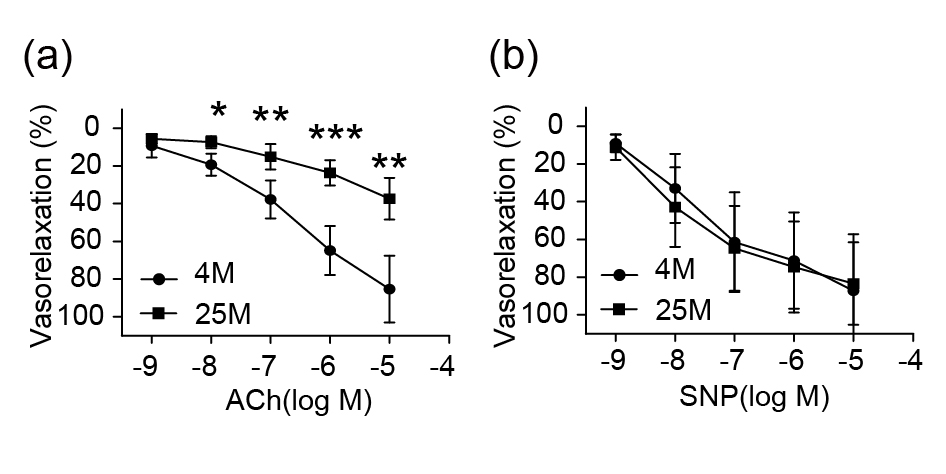

Supplement: Supplementary file 2 — Figure S2: Endothelium‐dependent and endothelium‐independent vasorelaxation in aged mice. (a) Endothelium‐dependent vasorelaxation responses to ACh were measured in aortic rings (n = 6 per group). Two‐way ANOVA. (b) Endothelium‐independent vasorelaxation responses to SNP were measured in aortic rings (n = 6 per group). Two‐way ANOVA. Data are presented as mean ± SD. *p < 0.05, **p < 0.01, ***p < 0.001. [file ACEL-25-e70652-s003.jpg]

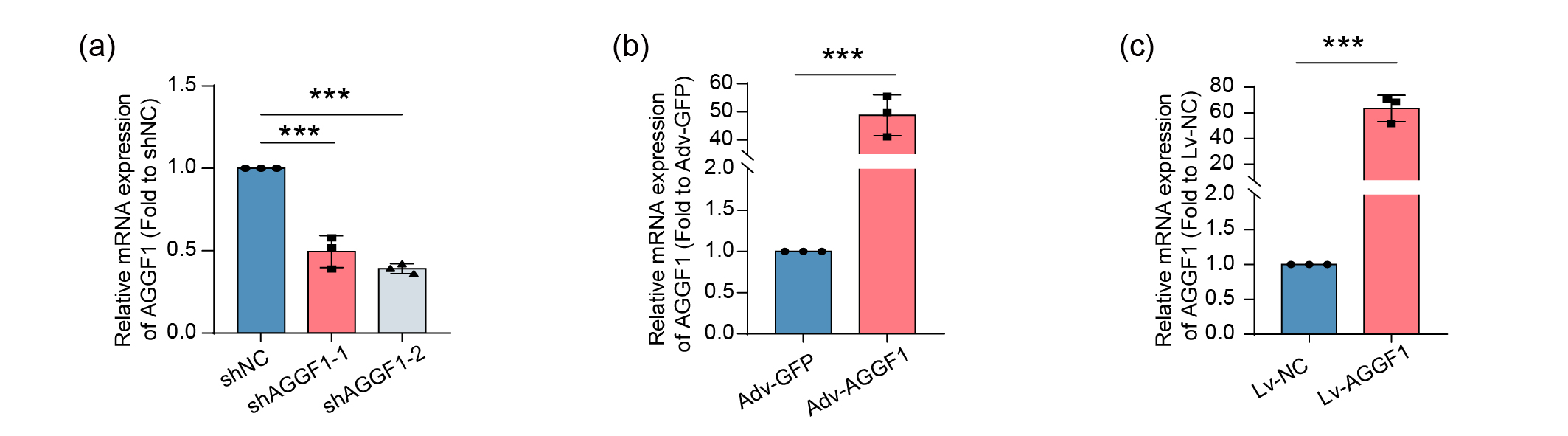

Supplement: Supplementary file 3 — Figure S3: Validation of AGGF1 overexpression and knockdown in HUVECs. (a) AGGF1 mRNA expression was markedly decreased in HUVECs infected with shAGGF1 lentivirus (n = 3 per group, 3 independent experiments). One‐way ANOVA. (b) AGGF1 mRNA expression was significantly increased in HUVECs infected with Adv‐AGGF1 adenovirus (n = 3 per group, 3 independent experiments). Two‐tailed Student's t‐test. (c) AGGF1 mRNA expression was significantly increased in HUVECs infected with Lv‐AGGF1 lentivirus (n = 3 per group, 3 independent experiments). Two‐tailed Student's t‐test. Data are presented as mean ± SD. ***p < 0.001. [file ACEL-25-e70652-s004.jpg]

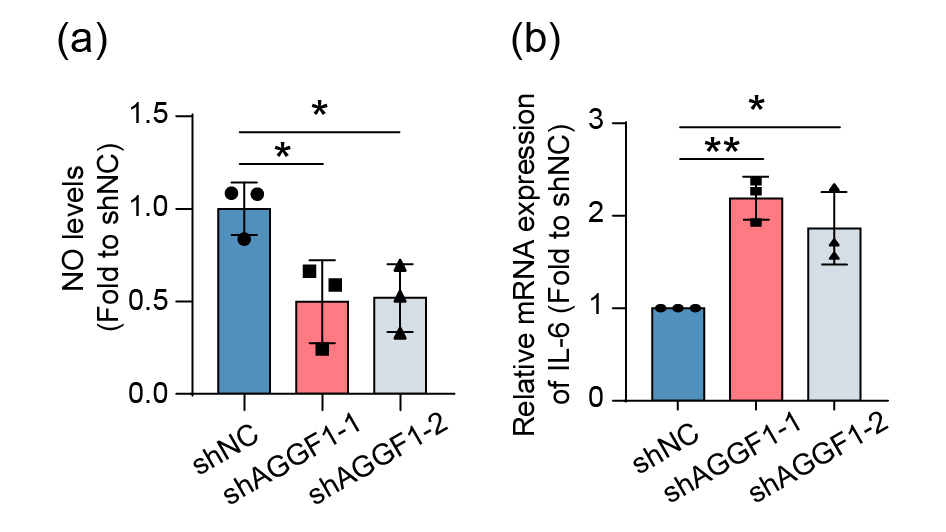

Supplement: Supplementary file 4 — Figure S4: AGGF1 knockdown reduces NO production and upregulates IL‐6 expression in HUVECs. (a) Quantification of NO concentration in the culture supernatant of HUVECs (n = 3 per group, 3 independent experiments). One‐way ANOVA. (b) IL‐6 mRNA expression was analyzed by qRT‐PCR (n = 3 per group, 3 independent experiments). One‐way ANOVA. Data are presented as mean ± SD. *p < 0.05, **p < 0.01. [file ACEL-25-e70652-s002.jpg]

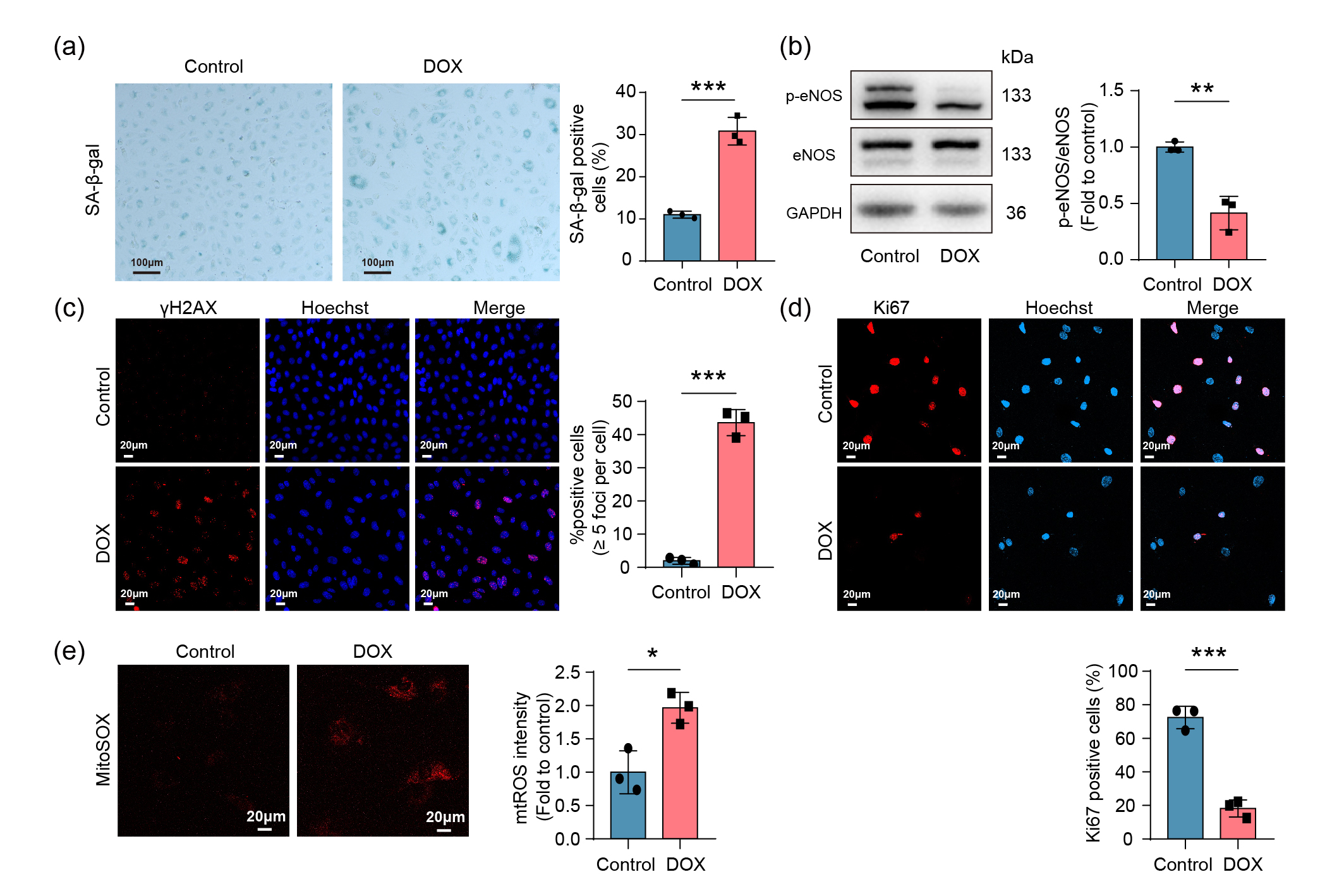

Supplement: Supplementary file 5 — Figure S5: DOX induces endothelial cell senescence. HUVECs were treated with 25 nM DOX for 24 h. (a) Representative images and quantification of SA‐β‐gal positive cells (n = 3 per group, 3 independent experiments), scale bar = 100 μm. Two‐tailed Student's t‐test. (b) Western blot analysis of eNOS and p‐eNOS (n = 3 per group, 3 independent experiments). Two‐tailed Student's t‐test. (c) Immunofluorescence staining for γH2AX (red) and Hoechst (blue) in HUVECs (n = 3 per group, 3 independent experiments), scale bar = 20 μm. Two‐tailed Student's t‐test. (d) Immunofluorescence staining for Ki67 (red) and Hoechst (blue) in HUVECs (n = 3 per group, 3 independent experiments), scale bar = 20 μm. Two‐tailed Student's t‐test. (e) Representative images of mtROS detected by MitoSOX staining, with quantitative analysis of relative fluorescence intensity (n = 3 per group, 3 independent experiments), scale bar = 20 μm. Two‐tailed Student's t‐test. Data are presented as mean ± SD. *p < 0.05, ***p < 0.001. [file ACEL-25-e70652-s008.jpg]

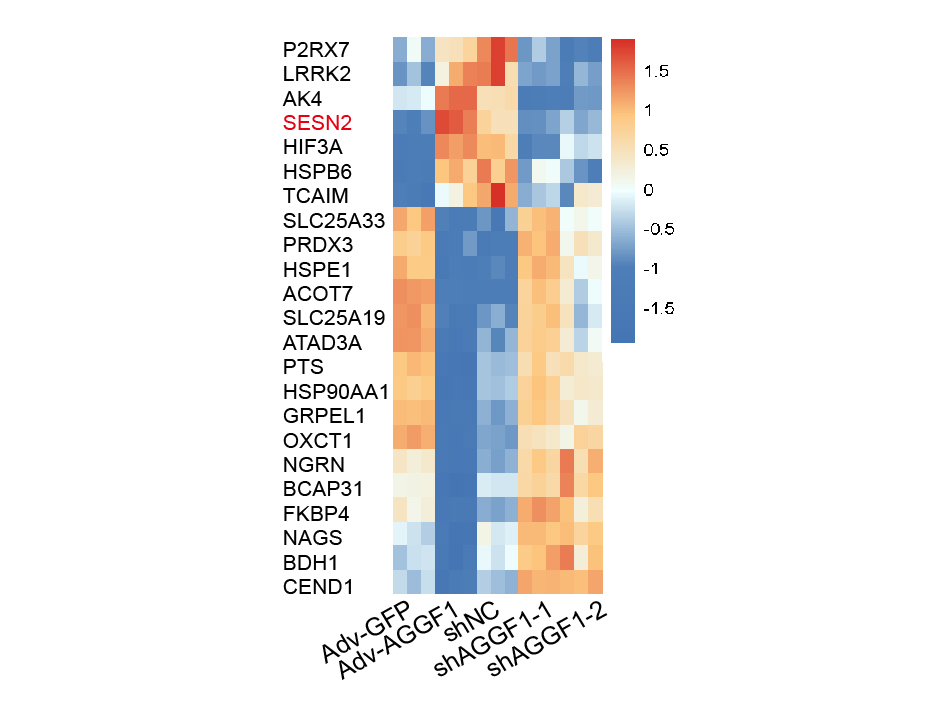

Supplement: Supplementary file 6 — Figure S6: Heatmap of mitochondrial pathway‐related genes co‐expressed with AGGF1. Among the 199 differentially co‐expressed genes, mitochondrial pathway‐related genes were identified by GO enrichment analysis. [file ACEL-25-e70652-s009.jpg]

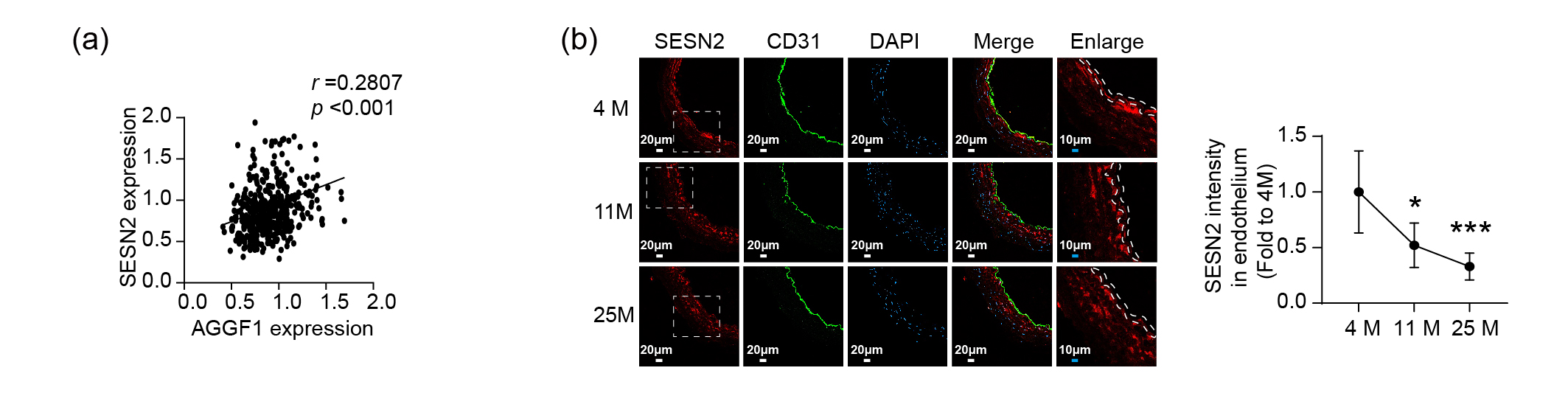

Supplement: Supplementary file 7 — Figure S7: SESN2 expression is positively correlated with AGGF1 in human vasculature and declines with age in mouse aortic endothelium. (a) Correlation analysis of SESN2 and AGGF1 expression in the human vascular tissues of elderly individuals from the ADEIP database. Spearman correlation analysis revealed a significant positive correlation (r = 0.2807, p < 0.001). (b) Immunofluorescence staining for SESN2 (red), CD31 (green), and DAPI (blue) in the aortic endothelium of young (4‐month‐old), middle‐aged (11‐month‐old), and aged (25‐month‐old) mice (n = 6 per group), white scale bar = 20 μm, blue scale bar = 10 μm. One‐way ANOVA. Data are presented as mean ± SD. *p < 0.05, ***p < 0.001. [file ACEL-25-e70652-s001.jpg]

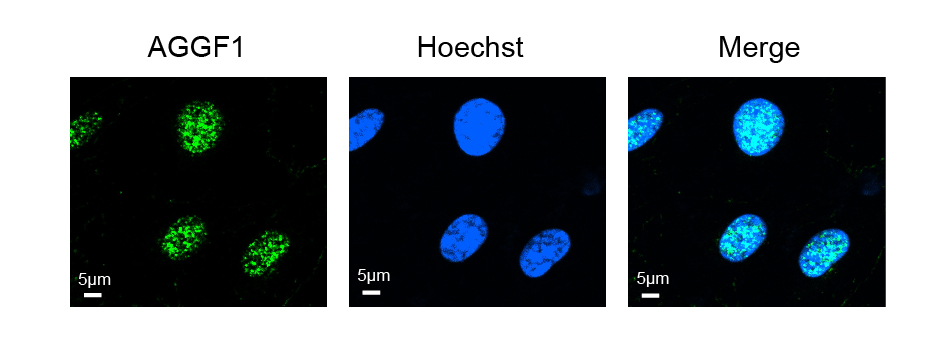

Supplement: Supplementary file 8 — Figure S8: AGGF1 exhibits nuclear localization in quiescent endothelial cells. Immunofluorescence staining for AGGF1 (green) and Hoechst (blue) in HUVECs, scale bar = 5 μm. [file ACEL-25-e70652-s005.jpg]

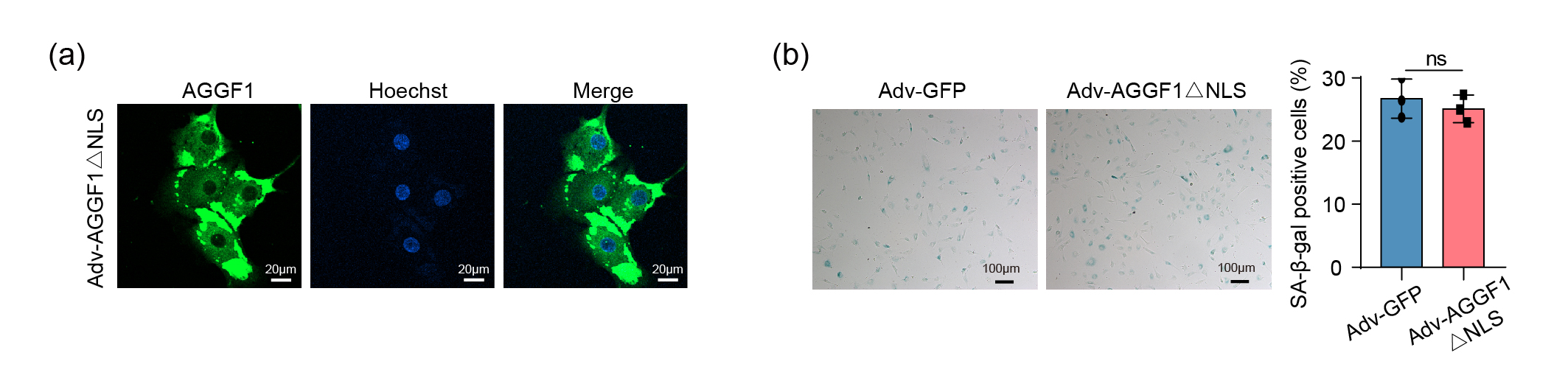

Supplement: Supplementary file 9 — Figure S9: Nuclear localization of AGGF1 is required for its protective effect against endothelial cell senescence. (a) Immunofluorescence staining for AGGF1 (green) and Hoechst (blue). The ΔNLS mutant protein was absent from the nucleus, scale bar = 20 μm. (b) Representative images and quantification of SA‐β‐gal positive cells (n = 3 per group, 3 independent experiments), scale bar = 100 μm. Two‐tailed Student's t‐test. Data are presented as mean ± SD. ns, not significant. [file ACEL-25-e70652-s006.jpg]

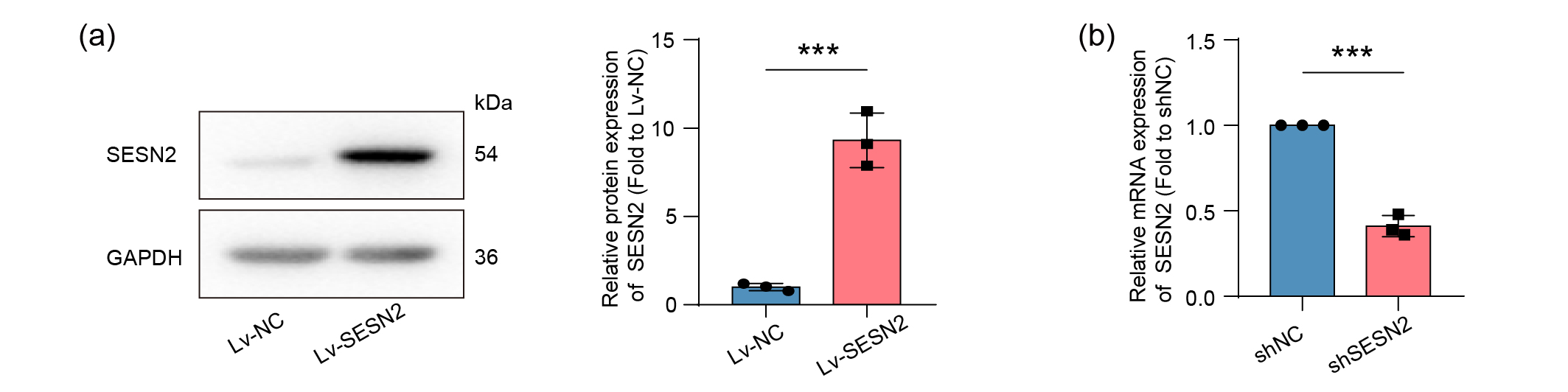

Supplement: Supplementary file 10 — Figure S10: Validation of SESN2 overexpression and knockdown in HUVECs. (a) SESN2 expression was significantly increased in HUVECs infected with SESN2 lentivirus (n = 3 per group, 3 independent experiments). Two‐tailed Student's t‐test. (b) SESN2 mRNA expression was significantly decreased in HUVECs infected with shSESN2 lentivirus (n = 3 per group, 3 independent experiments). Two‐tailed Student's t‐test. Data are presented as mean ± SD. ***p < 0.001. [file ACEL-25-e70652-s011.jpg]
